# Systematic estimation of biological age of *in vitro* cell culture systems by an age-associated marker panel

**DOI:** 10.3389/fragi.2023.1129107

**Published:** 2023-02-15

**Authors:** Christiane Hartmann, Luise Herling, Alexander Hartmann, Verena Köckritz, Georg Fuellen, Michael Walter, Andreas Hermann

**Affiliations:** ^1^ Translational Neurodegeneration Section “Albrecht-Kossel”, Department of Neurology, University Medical Center Rostock, Rostock, Germany; ^2^ Institute of Clinical Chemistry and Laboratory Medicine, University Medical Center Rostock, Rostock, Germany; ^3^ Institute for Biostatistics and Informatics in Medicine and Ageing Research, Rostock University Medical Center, Rostock, Germany; ^4^ Center for Transdisciplinary Neurosciences Rostock (CTNR), University Medical Center Rostock, Rostock, Germany; ^5^ Deutsches Zentrum für Neurodegenerative Erkrankungen (DZNE) Rostock/Greifswald, Rostock, Germany

**Keywords:** age marker, biological age - chronological age, age panel, skin fibroblast, progeria syndrome

## Abstract

Aging is a process that affects almost all multicellular organisms and since our population ages with increasing prevalence of age-related diseases, it is important to study basic processes involved in aging. Many studies have been published so far using different and often single age markers to estimate the biological age of organisms or different cell culture systems. However, comparability of studies is often hampered by the lack of a uniform panel of age markers. Consequently, we here suggest an easy-to-use biomarker-based panel of classical age markers to estimate the biological age of cell culture systems that can be used in standard cell culture laboratories. This panel is shown to be sensitive in a variety of aging conditions. We used primary human skin fibroblasts of different donor ages and additionally induced either replicative senescence or artificial aging by progerin overexpression. Using this panel, highest biological age was found for artificial aging by progerin overexpression. Our data display that aging varies depending on cell line and aging model and even from individual to individual showing the need for comprehensive analyses.

## 1 Introduction

Aging, usually described as a time-dependent functional decline (López-otín et al., 2013), is a physiological process that affects nearly all multicellular organisms. It is, however, the most important risk factor for the development of age-associated diseases (Rodgers et al., 2019). In general, aging includes processes that reduce health and survival of an individual (Fuellen et al., 2019). However, an individual’s chronological (the amount of years since birth) and biological age (reflecting actual physiological health state) can differ remarkably (Jylhävä, Pedersen, and Hägg, 2017; Kudryashova et al., 2020). In *in vitro* cell culture conditions, there are mainly two main contributors of the latter, which are the age of the donor at biopsy as well as the replicative aging/senescence that the respective cells have undergone so far. If we wish to consider physiological health state, the age of the donor at biopsy should be his or her estimated biological age. In practice, however, this biological age is rarely available and it must be substituted by the donor’s chronological age, which we call “donor age” in the following. In principle, the two main contributors cannot easily be distinguished with respect to their effects on the physiological “health” state of the cells. Thus, for the purpose of this paper, we define the age of cells in a cell culture (that is, their biological age) as the donor age plus the effects of continued culturing expressed as a time period in terms of donor age. This biological age can be *estimated* by biomarkers (which we call age markers) and it corresponds to a physiological “health” state which is predictive of the future behaviour of the cells. This affords a more precise description of the respective cell models used in scientific studies concerning their biological age, before they are used for experiments, which is recommended for a better characterization of the cells.

Aging processes have been studied in a variety of model systems. In addition to the various mouse models, *in vitro* cell cultures are the most commonly used systems. There are a variety of cell models for aging and all types of (age-related) diseases including simple cell models with heterogeneous expression systems (HeLA, HEK, etc.), patient-derived primary cells (e.g., fibroblasts) as well as induced-pluripotent stem cell (iPSC) models. Since the discovery of the latter, cell models of disease and/or aging have become of even greater interest. While these technologies enable to investigate patient-derived cell models of so far not available origins (e.g., neurons), these seem to significantly alter general aspects of cell physiology including their biological age. Especially in research on age-related diseases, it is therefore important to also consider the effects of such rejuvenation, e.g., by the process of iPSC generation, on various age markers.

An age marker is a measurable characteristic for estimating biological age or a related condition (Atkinson et al., 2001) in a biological system/organism. In the past, numerous suggestions for age markers have been made for the estimation of the biological age and its deviation from chronological age.

There are different aging hallmarks, and for some of them there are established markers to characterize biological age. For example, López-Otín described nine hallmarks of aging in three different main categories (López-otín et al., 2013). The primary hallmarks of aging (genomic instability, telomere attrition, epigenetic alterations, impaired proteostasis) are considered to be the main causes of cellular damage associated with rising age. These primary hallmarks lead to the antagonistic hallmarks of aging (deregulated nutrient sensing, mitochondrial dysfunction and cellular senescence), reactions that initially mitigate the damage but—if chronically present—can become harmful themselves. Finally, there are the integrative hallmarks of aging (stem cell exhaustion, altered intercellular communication), which are results of the previous two groups that are ultimately responsible for the loss of function associated with aging. These aging hallmarks can be investigated by various markers and methods (for summary see Supplementary Table S1).

Many putative age markers have thus been described and the age markers investigated in different studies often differ significantly (Hartmann et al., 2021). For this reason, a comparison of these studies among each other is often difficult and a specific comparison e.g., between different cell lines and conditions (rejuvenated cells and aged cells) is even more difficult. Since the latter can also affect age markers differentially, it seems meaningful to rely on a broader spectrum of age markers to better examine the variety of components of the aging process especially in *in vitro* cell culture systems.

The epigenome, especially DNA methylation, thereby is the most often reported singular factor to quantify an individual’s age. DNA methylation marks are the basis of so called “aging clocks” and often correlate well with the chronological age of the donor from which the tissue was derived. Furthermore, there is evidence that they can be used to estimate biological age. In 2013, Hovarth developed one of the first “aging clocks” that allows to estimate the chronological age by DNA methylation markers, for many tissues and some cell types. In this study, a collection of available DNA methylation data sets was used to define and evaluate a specific age predictor, based on CpG islands whose DNA methylation levels correlate with the chronological age of individuals, referred to as DNAm age. The difference between the predicted chronological age and the actual chronological age is then considered to be an estimate of biological age. Accordingly, DNAm age showed high accuracy in estimating chronological age (Horvath, 2013). Of note are, however, the reported exceptions: amongst others, high accuracy was not found for dermal fibroblasts in general as well as B cells from patients suffering from progeria syndromes. The issue was resolved in a follow up study, by the so called “Skin and blood clock” (Horvath et al., 2018). Another methylation clock that predicts human age by using the Illumina Infinium HumanMethylation450 assay in human whole blood samples additionally showed an influence of sex and genetic variants on the DNAm age, but also that aging rates are different in different tissues (Hannum et al., 2013).

Using the so called “CultureAge” score, it was shown that *in vitro* cellular aging resembled tissue aging *in vivo*. This study established an algorithm using replicative aged mouse embryonic fibroblasts and subsequently validated it in multiple murine tissues. Interestingly, it was sensitive to detect rejuvenation during cellular reprogramming, but is was reported that this progressive kind of cellular aging is different from non-replicative senescence (e.g., induced by etoposide treatment or gamma irradiation) and reported a lack of correlation of SA-β-Galactosidase with the CultureAge score (Minteer et al., 2022). Kabacik and colleagues also reported that epigenetic aging—using the skin and blood clock—is different from cellular senescence, telomere attrition and genomic instability, but is influenced by nutrient sensing and mitochondrial activity (Kabacik et al., 2022). Based on single cell methylation data, Trapp and colleagues established the epigenetic clock “scAge” using different murine tissues and cells, capable of representing chronological age at single cell level, and their algorithm is independent of which CpGs are covered in each cell. Nevertheless, the study showed remarkable heterogeneity among cells. Whether the use of this epigenetic clock is applicable to cultured cells, needs to be clarified in further studies (Trapp, Kerepesi, and Gladyshev, 2021). Fleischer et al. claimed to be able to predict biological age by analyzing the transcriptome of human fibroblasts. They generated a big dataset of genome-wide RNA-seq profiles of human fibroblasts to estimate the biological age of these cells. The predicted age correlated with the chronological donor age (Fleischer et al., 2018).

While these “-omic” approaches are very useful in generating hypotheses, they lack information about how cells behave in cell culture and thus lack the biological meaning of the respective changes. Furthermore, these investigations are often expensive and need specific equipment not necessarily available. Additionally, these have reported quite a significant diversity of aging and senescence processes, partly lacking correlation to classical age markers such as SA-β-galactosidase.

**FIGURE 1:**
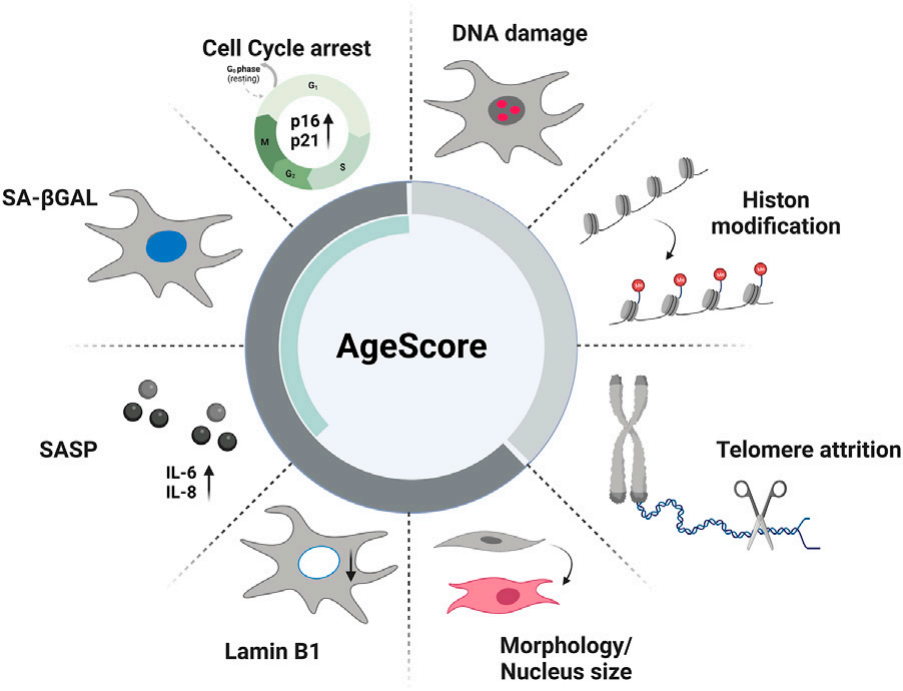
Age markers of proposed AgeScore. The here proposed panel contains the following age markers: primary aging markers (DNA damage, Histone modification, telomere attrition) and antagonistic aging markers (cell cycle arrest, senescence-associated βGalactosidase (SA-βGAL), senescence-associated secretory phenotype (SASP), Lamin B1 expression, Morphology) that include typical markers of cellular senescence (green bar).

Thus, our overall aim was to allow comparison of different aspects of aging in *in vitro* cell culture models with different donor background, different donor ages and accelerating age diseases, including different modes of aging e.g., replicative and artificial aging. Therefore, we first made an extensive literature search to identify established markers that are most likely relevant in cellular aging processes. We chose often-used markers from the primary and antagonistic hallmarks of aging as they are quantifiable by *in vitro* assays to investigate whether they can clearly depict the different aspects of aging. Subsequently, we developed a novel biomarker-based panel, consisting of well established classical age markers, to estimate the biological age of different cell culture aging systems that additionally can be used in standard cell culture laboratories (Figure 1). We hypothesized that this panel of age markers would better account for differences between individual cell lines and aging models and individuals than single markers. After confirming the aging-relevance of the single age markers we selected, we propose a scoring system, referred to as AgeScore, to calculate an individual absolute value as an estimate of the biological age of different human fibroblasts. Further validation of this proposed scoring system took place in human fibroblasts with replicative senescence and artificial aging by overexpression of progerin. Finally, we tested whether our aging panel is applicable in typical aging diseases (premature aging syndromes Hutchinson-Gilford-Progeria syndrome, HGPS, and Werner Syndrome, WS). Conclusively, we propose an age score consisting of different well established markers representing aging and the consequences of aging as follows: 1) markers of the primary hallmarks of aging (DNA damage, telomere attrition, Histone modification) displaying the cellular damage, cell division and methylation status of the cells and 2) markers of the antagonistic hallmarks of aging (cellular senescence [cell cycle arrest, SA-β-Gal expression, activation of SASP], change of morphology, decrease in Lamin B1 expression) displaying how much the primary damage has already influenced cell homeostasis which display together a fundamental AgeScore estimating the biological age of cells in cell culture systems.

## 2 Materials and methods

### 2.1 Cell culture

Human skin fibroblasts from apparently healthy young, mid-age and old as well as diseased (Progeria) donors were purchased from Coriell Institute or prepared in our lab as described before (Naumann et al., 2018; Pal et al., 2018, 2021). Cells were cultured in Dulbecco-modified Eagle’s medium (DMEM-Glutamax, ThermoFisher Scientific, #10569010) supplemented with 15% fetal bovine serum (FBS superior, ThermoFisher Scientific, #10270106) and penicillin/streptomycin (100 μg/mL, Fisher Scientific, #15140-122) on ventilated culture flasks (75 cm^2^, Fisher Scientific, #658175) at 37°C and 5% CO_2_. Fibroblasts were subcultured after trypsinisation (0.25% trypsine-EDTA, ThermoFisher Scientific, #25200-056). Culture medium was changed every 3–4 days.

### 2.2 Determination of population doubling level (PDL)

For determination of PDL, human fibroblasts were seeded into 6-well-plates (Sarstedt, #833.920) at a density of 2x10^5^ per well. When fibroblasts reached confluency, cells were counted and again seeded at a density of 2x10^5^. This was continued over 75–150 days. PDL was calculated as PDL = 3.2*(log_harvested cells_—log_seeded cells_) + actual passage number.

### 2.3 Immunofluorescence staining

Fibroblasts were seeded at a density of 2.5x10^4^ per well in an 8-well µ-Slide (ibidi, #80806) and incubated for 48 h. Cells were fixed with 4% PFA at 37°C for 20 min followed by permeabilization with 0.2% of TritonX and blocked with Blocking Buffer (Pierce Protein-Free T20 (TBS) Blocking Buffer, ThermoFisher Scientific, #37571) at RT for 1 h. Primary antibodies (Anti-phospho-Histone H2A.X (Ser139), #05-636-25ug, 1:500, Millipore; Anti-Histone H3 (di methyl K9), #ab1220, 1:500, abcam; Anti-Lamin B1, #ab16048, 1:500, abcam; Anti-Vimentin, #PA1-16759, 1:300, Thermo Fisher Scientific) were incubated overnight at 4°C. After washing, secondary antibodies (Goat anti-Mouse IgG (H + L) Highly Cross-Adsorbed Secondary Antibody, Invitrogen; Goat anti-Rabbit IgG (H + L) Highly Cross-Adsorbed Secondary Antibody, Invitrogen) were incubated for 90 min. Finally, after repeated washing, cells were mounted with 4.6-diamidino-2-phenylindole (DAPI) Fluoromount-G (Southern Biotech, #0100-20) and images were taken with a LSM900 confocal microscope (Zeiss). Analysis was done with Fiji software. γH2A.X foci were counted per nucleus (threshold: 20-200; size: 5). Corrected total cell fluorescence (CTCF) was calculated as following: (CTCF) = Integrated Density—(Area of selected Cell/Region * Mean Grey Value of background readings). Nucleus size was determined with area (pixel units) of nucleus with Fiji software.

### 2.4 Telomere length measurement *via* monochromal multiplex qPCR (MM-qPCR)

A standard extraction kit (DNeasy Blood and Tissue Kit, Qiagen, Cat. # 69504) was used for DNA extraction. Mean telomere length was determined using the modified MM-qPCR as described previously (Cawthon, 2009). DNA samples (20 ng/μL) and a reference DNA standard (0.137–100 ng/μL) were assayed in triplicates on different plates and the average of three measurements was used to report the mean telomere length for each sample. A non-template control (water) and a positive control (human leukemia cell line 1301 DNA) were prepared in duplicates and run on every plate. The standard includes DNA samples of 352 healthy donors, with an average age of 40.14 years (18–70 years old; 38.35% males and 61.65% females). The assay was performed using a BioRad CFX384 real-time C1000 thermal cycler with the following profile: 1 cycle of 15 min at 95°C; 2 cycles of 15 s at 94°C, 1 cycle of 15 s at 49°C; 40 cycles of 15 s at 94°C, 1 cycle 10 s at 62°C, 1 cycle 15 s at 72°C with T signal acquisition, 10 s at 85°C, and 15 s at 89°C with signal acquisition. PCR reagents were used at the following final concentrations: 1 U titanium Taq DNA polymerase per reaction with provided titanium Taq PCR buffer (Cat. # 639208), SYBR Green I (Invitrogen, #S7563), 0.2 mM of each dNTP, 1 mM DTT, 1 M betaine, 900 nM of each telomere primer (*Telg, Telc*) and 300 nM of each single copy gene primer (*ALBu, ALBd*). Following primer sequences were used: *Telg 5′ACA​CTA​AGG​TTT​GGG​TTT​GGG​TTT​GGG​TTT​GGG​TTA​GTG​T′3*; *Telc 5′TGTTAGGTATCCCT ATC​CCT​ATC​CCT​ATC​CCT​ATC​CCT​AAC​A′3; Albu 5′CGG​CGG​CGG​GCG​GCG​CGG​GCT​GGG​CGG​AAA​TGC​TGC​ACA​GA ATCCTTG′3; Albd 5′GCC​CGG​CCC​GCC​GCG​CCC​GTC​CCG​CCG​GAA​AAG​CAT​GGT​CGC​CTG​TT′3*. Ratio of telomere to single-copy gene content (TLR) is taken as relative measurement of telomere length and expressed in arbitrary units. The intra-assay coefficients of variation were <0.3 for all samples.

### 2.5 Quantification of p21 and p16 expression levels

Total RNA was isolated using the “Quick RNA Miniprep” Kit (Zymo Research, #R1054) according to the manufacturer’s protocol and cDNA was generated from 200 ng isolated RNA with High-Capacity cDNA Reverse Transcription Kit (Thermo Fisher Scientific, #4368814). mRNA expression levels were determined using Rotor Gene (QIAGEN) with Rotor-Gene SYBR® Green PCR Kit (QIAGEN, #204074) using the following primers: *CDKN1A(p21)*-Fwd *5′-GAC​ACC​ACT​GGA​GGG​TGA​CT-3′, CDKN1A(p21)-*Rev *5′-CAGGTCCACATGGTCT TCCT-3′, CDKN2A(p16)*-Fwd *5′-CTC​GTG​CTG​ATG​CTA​CTG​AGG​A-3′, CDKN2A(p16)*-Rev *5′-GGTCGGCGCAGTT GGGCTCC-3′*. qPCRs were performed in duplicates for each sample (*n* = 1 correspond to 2 replicates). Data sets were normalized relative to *GAPDH* (*GAPDH*-Fwd *5′- GTC​TCC​TCT​GAC​TTC​AAC​AGC​G-3′, GAPDH*-Rev *5′- ACC​ACC​CTG​TTG​CTG​TAG​CCA​A-3′*) using delta-delta-CT method.

### 2.6 Senescence-associated β-galactosidase (SA-βGAL)-Assay

Activity of SA-β-Gal was determined using the “Senescence β-Galaktosidase staining” Kit (Cell signaling, #9860) according to the manufacturer’s protocol and referred to as X-Gal staining in the figures. Cells with cytoplasmic staining were scored as positive. Relative amount to total cell numbers was quantified.

### 2.7 IL-6 and IL-8 medium concentration

ELISA from cell culture supernatants was performed using “Human IL-6/Interleukin-6 ELISA Kit PicoKine™” (BosterBio, #EK0410) or “Human IL-8/Interleukin-8 ELISA Kit PicoKine™” (BosterBio, #EK0413) according to the manufacturer’s recommended procedures. ELISA were performed in duplicates for each sample (*n* = 1 correspond to 2 replicates).

### 2.8 Statistics

Statistical analyses were performed using GraphPad Prism 8 (LaJolla). Experimental groups were compared using one-way-ANOVA (followed by Dunnett’s multiple comparison test). Statistical significance was set up at *p*-values <0.05 (∗), <0.01 (∗∗), <0.001 (∗∗∗). Data were plotted using GraphPad Prism 8 (LaJolla) showing mean and standard error of the mean.

## 3 Results

### 3.1 Single age markers yield heterogeneous results in human fibroblasts of healthy donors with different ages

**FIGURE 2:**
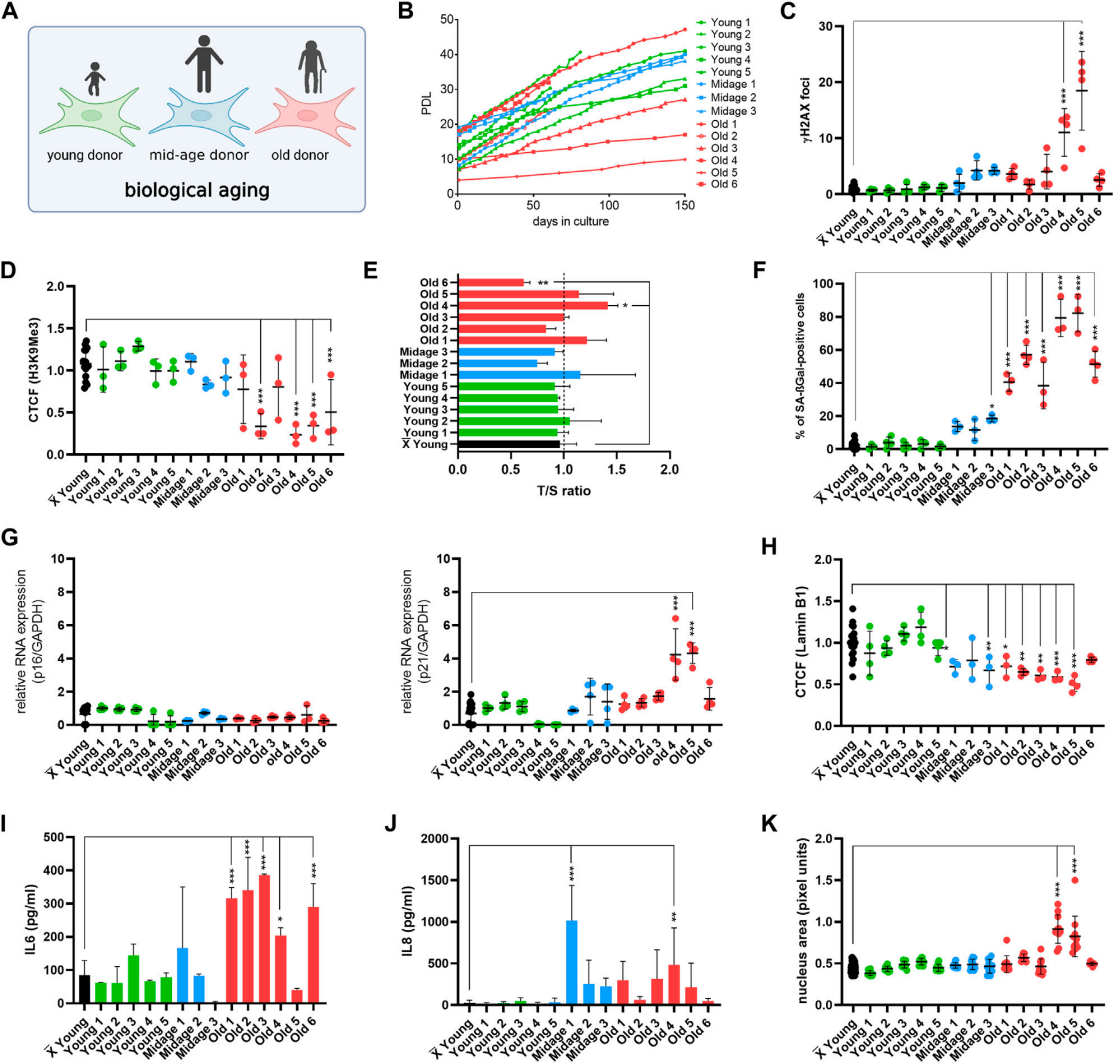
Establishment of the age marker-based panel in human fibroblasts of young, mid-age and old donors. **(A)** Cell Model. For initial experiments, fibroblasts with different donor age was used. **(B)** Replicative potential of human fibroblasts. Cells were cultured under stable conditions (37°C, 5% CO_2_) in DMEM medium (with 15% FBS, 1% Pen-Strep). PDL was observed for max. 150 days. Fibroblasts from young (Young 1–5) and mid-age (Midage 1–3) donors display healthy growth. In contrast, fibroblasts from old donors (Old 3, Old 4, Old 5 and 6) showed slower growth except for Old 1 and Old 2. **(C–K)** X̅ = Mean value of all young donor fibroblasts. **(C)** Amount of γH2A.X foci per cell examined by IF staining to investigate DSBs in human fibroblasts. Cells of very old individuals (Old 4 and 5) displayed an increase in γH2A.X foci in comparison to the mean of all young donors. Mid-age and old (Old 1, Old 2, Old 3 and Old 6) donor fibroblast showed no DSBs. [*n* = 3–4 (each n tested >15 cells), mean ± SEM,*p < 0.05, **p < 0,001, ***p < 0.0001, one-way ANOVA] **(D)** CTCF of H3K9Me3 in human fibroblasts. Donor fibroblasts from old individuals (Old 2, Old 4, Old 5 and Old 6) displayed a decrease in CTCF of H3K9Me3 compared to the mean of young donors. There were no changes in CTCF of H3K9Me3 in mid-age cells (Midage 1–3). [*n* = 3–4 (each n tested >15 cells), mean ± SEM, *p < 0.05, **p < 0.001, ***p < 0.0001, one-way ANOVA] **(E)** Telomere length was measured by MM-qPCR in comparison to a standard of healthy mixed aged individuals. In comparison to grouped young donor cells, Old 6 displayed a shortening of telomere length. [*n* = 3, mean ± SEM, *p < 0.05, **p < 0.001, ***p < 0.0001, one-way ANOVA] **(G)** RNA expression levels measured by qRT-PCR of p21 and p16 was examined to investigate activation of cell cycle arrest. Old 4 and Old 5 displayed an increase in RNA expression level of p21. All other cells did not show altered RNA expression levels. [*n* = 3-4, mean ± SEM *p < 0.05, **p < 0.001, ***p < 0.0001, one-way ANOVA] **(F)** Quantification of SA-βGAL positive cells by X-Gal staining. In comparison to grouped young donor cells, Midage 3 and all old human fibroblasts (Old 1–6) displayed a significant increase of SA- βGAL positive cells. [*n* = 3–4 (each n tested >15 cells), mean ± SEM, *p < 0,05, **p < 0.001, ***p < 0.0001, one-way ANOVA] **(H)** CTCF of Lamin B1 expression. A significant decrease in Lamin B1 expression could be observed in mid-age (Midage 1 and Midage 3) and old (Old 1–5) cells compared to the mean of young donor fibroblasts. [*n* = 4 (each n tested >15 cells), mean ± SEM, *p < 0.05, **p < 0.001, ***p < 0.0001, one-way ANOVA] **(I, J)** Determination of cytokine secretion (IL-6, IL-8) in conditioned medium by ELISA. In comparison to grouped young donor cells, old donor fibroblasts (Old 1, Old2, Old 3, Old 4, and Old 6) showed an increase in levels of IL-6. An increase in concentration with respect to IL-8 could be observed in Midage 1 and Old 4 in comparison to grouped controls. [*n* = 3-4, mean ± SEM, *p < 0.05, **p < 0,001, ***p < 0.0001, one-way ANOVA] **(K)** Change in nucleus morphology shown as nucleus area (pixel units) in human fibroblast. Nucleus area is significantly increased in very old donor fibroblasts (Old 4 and 5) compared with grouped young controls. [*n* = 6–8 (each n tested >15 cells), mean ± SEM *p < 0.05, **p < 0.001, ***p < 0.0001, one-way ANOVA].

We first wanted to analyze how selected age markers relate to chronological donor age. Therefore, we performed a systematic comparison of the selected age markers, considering these one by one as single markers, in human fibroblasts from 14 donors from apparently healthy young (2–9 years), mid-age (34–48 years), and old individuals (78–96 years) (Figure 2A; Table 1). Both male and female donors were included. Furthermore, we examined the youngest available cell culture passages of each fibroblast line. To observe growth rates of cells, we investigated the population doubling level (PDL) under stable conditions (37°C, 5% CO_2_) (Figure 2B). Primary cells from young (Young 1–5) and mid-age (Midage 1–3) donors showed healthy exponential growth. In contrast, fibroblasts from old (Old 3) and very old donors (Old 4, 5 and 6) displayed slower growth except for the fibroblasts Old 1 and 2, which resembled young donor cells in terms of growth (Figure 2B).

**TABLE 1:** Characteristics of human fibroblasts used in this study.

Primary cells	Alias (Coriell/PRF ID)	Age at biopsy	Sex	Youngest passage available	Source
Apparently healthy	Young 1 (AG07095)	2	Male	6	Coriell
	Young 2 (GM00969)	2	Female	13	Coriell
	Young 3 (GM05565)	3	Male	3	Coriell
	Young 4 (GM00498)	3	Male	10	Coriell
	Young 5 (GM00038)	9	Female	11	Coriell
	Mid-age 1	34	Male	5	Own lab
	Mid-age 2 (GM01653)	37	Male	14	Coriell
	Mid-age 3	48	Female	15	Own lab
	Old 1 (GM09918)	78	Male	15	Coriell
	Old 2 (GM03525)	80	Female	9	Coriell
	Old 3 (GM01706)	82	Female	5	Coriell
	Old 4 (AG09602)	92	Female	9	Coriell
	Old 5 (AG04059)	96	Male	7	Coriell
	Old 6 (GM00731)	96	Male	13	Coriell
HGPS	HGPS 1 (HGADFN003)	2	Male	13	Progeria research foundation
	HGPS 2 (HGADFN188)	2	Female	16	Progeria research foundation
WS	WS 1 (AG03141)	30	Female	14	Coriell
	WS 2 (AG06300)	37	Male	5	Coriell

Next, the primary hallmarks of aging (DNA damage, telomere attrition and histone modification) were examined. DNA damage is a driving force of aging because it has a number of molecular consequences that are also hallmarks of aging, such as genomic instability, telomere attrition, epigenetic changes, and/or impaired mitochondrial function (Schumacher et al., 2021). To analyze the number of DNA double-strand breaks (DSBs), we examined the number of γH2A.X foci by immunofluorescence staining (Figure 2C). Human fibroblasts from very old individuals (Old 4 and 5) showed significant higher amounts of DSBs compared with the average number of DSBs in young individuals. We next examined histone modification by immunofluorescence staining and quantifying the corrected total cell fluorescence (CTCF) of H3K9Me3 (Figure 2D). The heterochromatin loss model of aging assumes that heterochromatin domains which are formed early in embryogenesis decrease during aging and contribute to a global loss of heterochromatin-induced gene silencing, resulting in aberrant gene expression patterns (Villeponteau, 1997). In our study, we observed no changes in mid-age (Midage 1–3) but a decrease in H3K9Me3 expression in old donor fibroblasts (Old 2, Old 4, Old 5, and Old 6). In addition, we measured telomere length by monochromatic multiplex qPCR (MM-qPCR) (Figure 2E). Telomeres are specialized chromatin structures at the end of eukaryotic chromosomes that serve to protect chromosome ends. Telomere shortening with aging is observed in most human tissues in which it has been tested (Lange, 2005) and is one of the best understood mechanisms limiting the growth of normal cells in culture, a phenomenon referred to as “replicative senescence” (Collado, Blasco, and Serrano, 2007). Compared to the mean of young donor fibroblasts (Young 1–5), Old 6 was the only cell line exhibiting significant telomere shortening.

Following, single antagonistic age markers were systematically investigated, including induction of cell cycle arrest, expression of SA-βGal, activation of senescence-associated secretory phenotype (SASP), Lamin B1 expression and morphological changes (i.e., size of nucleus). Cell cycle arrest in senescence is largely mediated by activation of one or both of the tumor suppressor pathways p53/p21 and p16/pRB (Kumari and Jat, 2021). These two pathways are very complex, involving many upstream regulators and downstream effectors, as well as several side branches (Chen et al., 2002; Rovillain et al., 2011). Our readout for cell cycle arrest were RNA expression levels of p21 and p16 (Figure 2G). Old 4 and Old 5 showed a raise in RNA expression level of p21. Otherwise, none of the human fibroblasts showed altered RNA expression levels of p21 or p16. Subsequently, we determined the number of cells positive for SA-βGAL, one of the most used markers of cellular senescence. The lysosomal hydrolase β-galactosidase cleaves terminal β-galactose from compounds such as lactose, keratin sulfates, sphingolipids, etc. It is present in almost all tissues. The activity of this enzyme increases with development of cellular senescence (Dimri et al., 1995). We determined the amount of SA-βGAL positive cells (Figure 2F). In comparison to the mean of young donor cells (Young 1–5), old human fibroblasts (Old 1–6) displayed a significant increase of SA- βGAL positive cells, while in mid-age donors, only Midage 3 showed a significant increase in SA- βGAL positive cells. Following, we investigated expression levels of nuclear protein Lamin B1 by determining the CTCF (Figure 2H). Lamin B1 expression decreases with age and is considered as an age marker (Wang et al., 2017; Kristiani, Miri, and Youngjo, 2020). Recent studies have shown that the nuclear lamina regulates both the organization of three-dimensional chromatin structure at the nuclear periphery (Yue, Zheng, and Zheng, 2019) and gene expression, e.g., of inflammatory response genes (Shah et al., 2013). A significant decrease in Lamin B1 expression was observed in mid-age and old donor fibroblasts (Midage 1, Midage 3, Old 1–5) compared with the mean of young donor cells. For studying SASP activation, reflecting senescent cells (Coppé et al., 2008), we examined secreted concentrations of two principle SASP factors, the inflammatory cytokines interleukin 6 (IL-6) and interleukin 8 (IL-8) in conditioned medium of donor cells by ELISA (Figures 2I, J). An increase in concentration of IL-6 was detected in old donor fibroblasts (Old 1-Old 4 and Old 6) in comparison to grouped controls. Additionally, Midage 1 and Old 4 displayed a significant increase in levels of IL-8. Furthermore, it is known that nucleus size changes with age and in pathological conditions (Capell et al., 2005; Glynn and Glover, 2005; Haithcock et al., 2005; Webster, Wikin, and Cohen-Fix, 2009). To study morphological changes occurring with aging or during pathogenesis of age-associated diseases, we examined the size of the nucleus using pixel area of DAPI staining (Figure 2K). Nucleus size was significantly enlarged only in very old human donor fibroblasts (Old 4 and Old 5) compared to grouped young controls (Heckenbach et al., 2022).

In summary, when systematically investigating above mentioned well known single age markers, there was a remarkable heterogeneity between single human donors of different ages and it might thus be hard to judge upon the biological age if only investigating one single aging marker alone. While SA-βGAL was the best single age marker to depict old donor age, it did not perfectly distinguish midage from young donor age. Therefore, we went on to develop a strategy to use above mentioned markers together in one panel to finally yield an integrated AgeScore result.

### 3.2 Calculation of proposed AgeScore correlates with donor age of examined human fibroblasts

**FIGURE 3:**
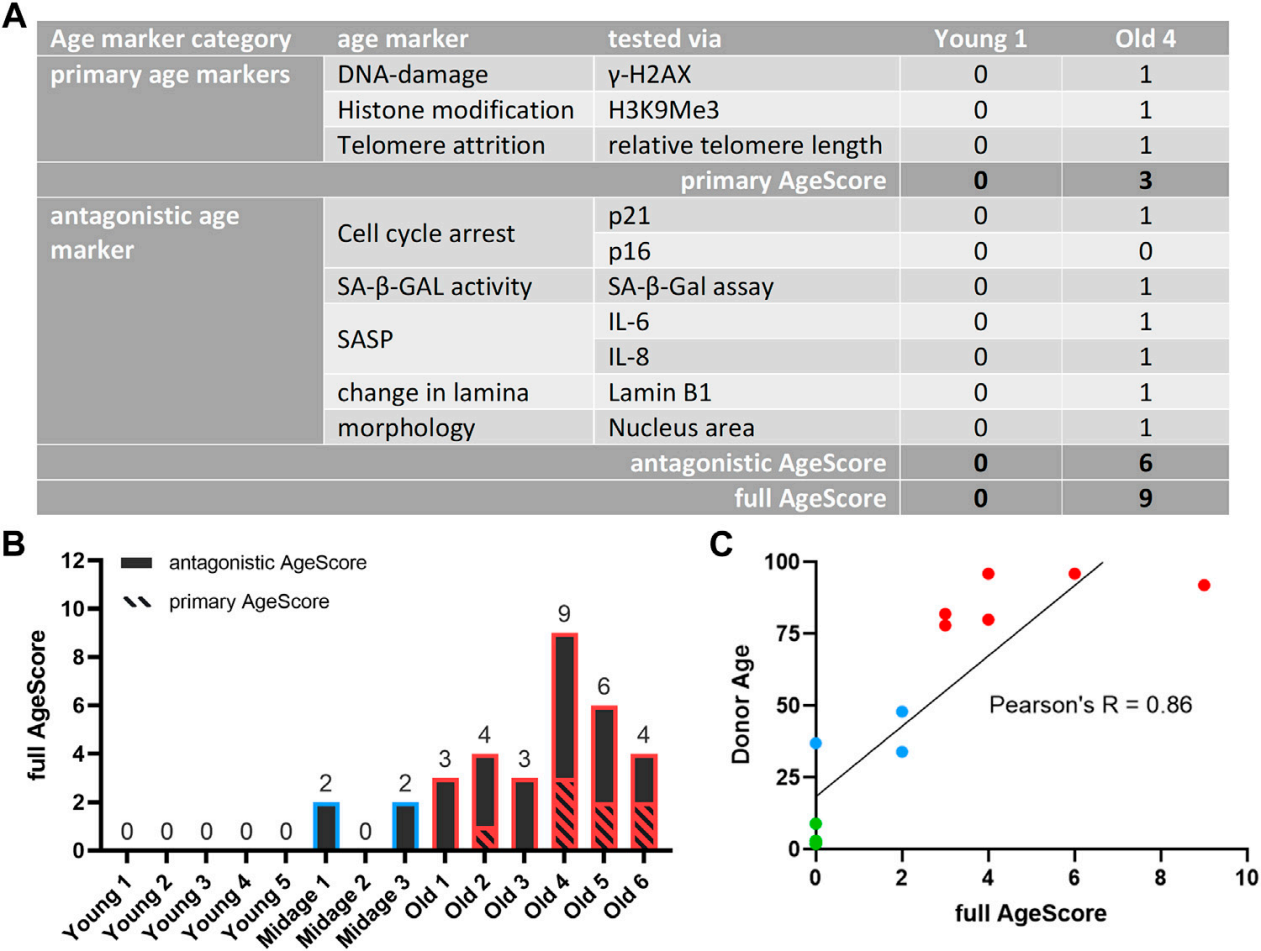
Calculated full AgeScore correlates with fibroblasts donor age. The here proposed full AgeScore (primary AgeScore + antagonistic AgeScore) correlates with donor age of examined human fibroblasts. **(A)** Calculation of proposed AgeScore for given examples (Young 1, Old 4). Each fibroblast tested receives a 0 for a non-significant age marker and a 1 for a significant age marker tested. These values are summed up to the primary AgeScore and the antagonistic AgeScore. The sum of both is the full AgeScore. **(B)** Calculated AgeScore for examined human fibroblasts displays an increase of the AgeScore with upcoming donor age. **(C)** Pearson correlation of full AgeScore and donor age of tested fibroblasts. The donor age correlates with our calculated AgeScore with a Pearson’s R of 0.86.

To take the individual cell line variabilities better into account we used above mentioned single markers to generate one panel with a single absolute value from the tested age markers and asked ourselves whether this value then depicts the chronological age of fibroblasts of different donor ages as good as or even better than single markers (Figure 3A). To create this score (hereinafter referred to as AgeScore) each primary cell culture system examined received an equal weight of “1” for a significant (positive) age marker result and a weight of “0” for each non-significant (negative) result. Subsequently, results of tested age markers were summed up to calculate the (A) primary AgeScore and (B) the antagonistic AgeScore. Finally, both scores together resulted in the (C) full AgeScore that was able to distinguish between young and old donor derived fibroblasts showing an increase with age (Figure 3B). For every used age marker we calculated Pearson’s correlation and most age markers correlate with the AgeScore (Supplementary Figure S1). We observed a very high correlation for the markers H3K9Me3, p21, nucleus size and SA-β-Gal and a high correlation for Lamin B1 and H2AX. However, there is a moderate correlation for telomere length and a low correlation for p16, IL6, and IL8. Whereby all young donor fibroblasts showed a full AgeScore of 0, only the antagonistic AgeScore increased in mid-age fibroblasts (antagonistic AgeScore = 2). In old donor fibroblasts, primary as well as antagonistic AgeScores increased and resulted in the highest full AgeScore calculated. The Pearson`s correlation of *R* = 0.86 (Figure 3C) demonstrated that our AgeScore tended to increase with donor age of examined human fibroblasts.

**FIGURE 4:**
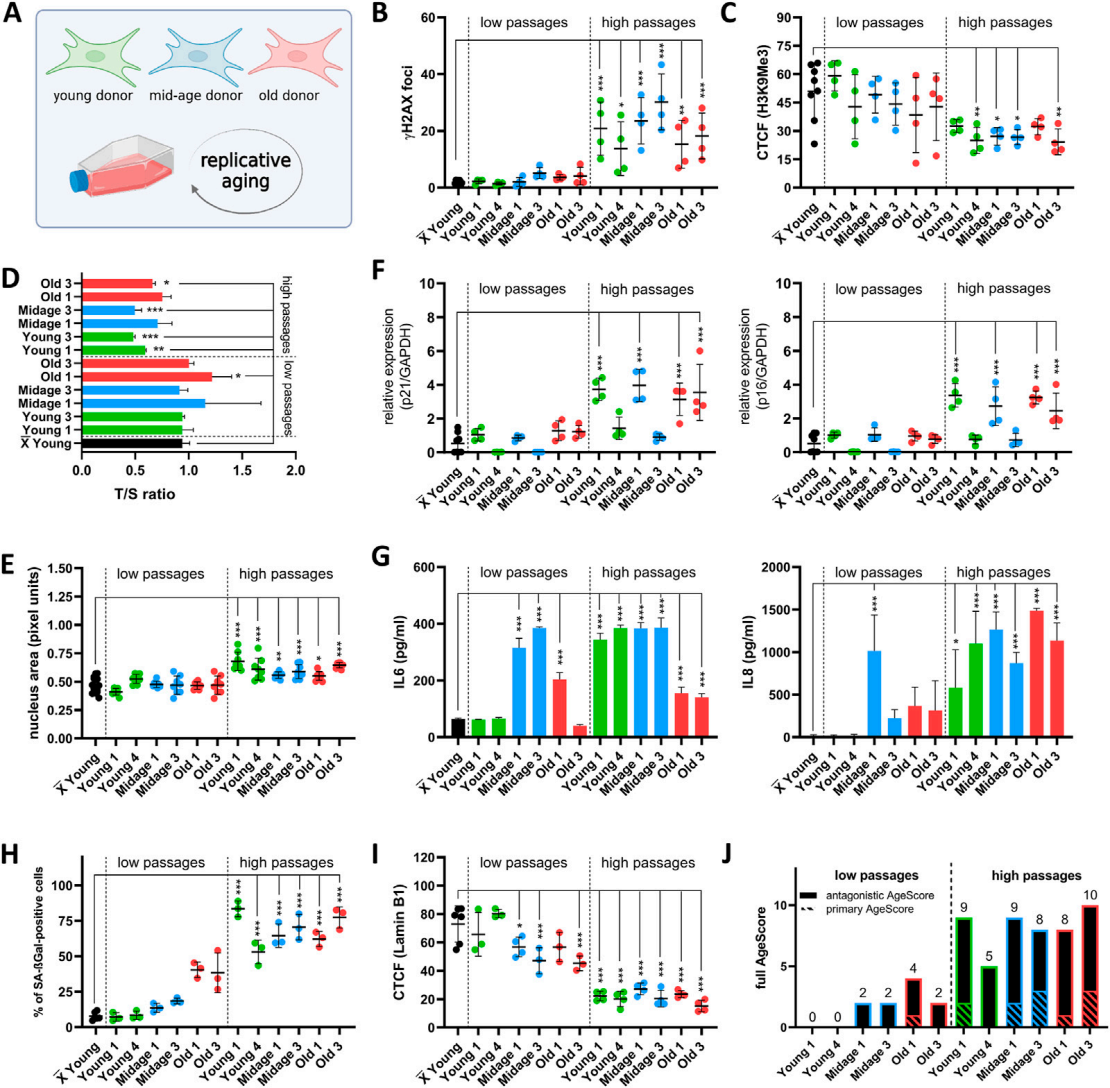
Examination of the AgeScore in human fibroblasts under replicative senescence. **(A)** Cell Model for validation of proposed AgeScore, fibroblasts from young, mid-age and old donors were replicative aged. **(B–I)** X̅ = Mean value of all young donors. **(B)** Quantification of γH2A.X in fibroblasts in high passages (*p* > 35) in comparison to the mean of all Young^low^. Amount of γH2A.X foci per cell increased in all cells in high passages. [*n* = 4 (each n tested >15 cells), mean ± SEM,*p < 0.05, **p < 0.001, ***p < 0.0001, one-way ANOVA] **(C)** Quantification of H3K9Me3 by IF staining. CTCF displayed a significant decrease of H3K9Me3 expression in high passages of Young 4, Midage 1, Midage 3 and Old 3 compared to grouped Young^low^. [*n* = 4 (each n tested >15 cells), mean ± SEM,*p < 0.05, **p < 0.001, ***p < 0.0001, one-way ANOVA] **(D)** Telomere length was measured by MM-qPCR in comparison to a standard of healthy mixed aged individuals. In comparison to grouped Young^low^, high passages of Young 1, Young 2, Midage 3 and Old 3 displayed a shortening of telomere length. [*n* = 3, mean ± SEM, *p < 0.05, **p < 0.001, ***p < 0.0001, one-way ANOVA] **(F)** RNA expression levels by qRT-PCR of p21 and p16. Cell cycle arrest was detected by upregulation of p21 and p16 in Young 1^high^, Midage 1^high^, Old 1^high^ and Old 3^high^ [*n* = 4, mean ± SEM *p < 0,05, **p < 0.001, ***p < 0.0001, one-way ANOVA] **(G)** Investigation of cytokine secretion (IL-6, IL-8) in conditioned medium by ELISA. SASP activation was elevated in all fibroblasts under replicative senescence in comparison to the mean of all Young^low^ [*n* = 3-4, mean ± SEM, *p < 0.05, **p < 0.001, ***p < 0.0001, one-way ANOVA] **(E)** Quantification of nucleus area by pixel units. Nucleus area was significantly increased in all high passages of human fibroblasts in comparison to grouped low passages of Young^low^. [*n* = 8 (each n tested >15 cells), mean ± SEM *p < 0.05, **p < 0.001, ***p < 0.0001, one-way ANOVA] **(H)** Quantification of SA-βGAL positive cells by X-Gal staining. Amount of positive SA-βGal stained cells was significantly increased in all cells in high passage in comparison to the mean of all Young^low^. [*n* = 4 (each n tested >15 cells), mean ± SEM, *p < 0,05, **p < 0.001, ***p < 0.0001, one-way ANOVA] **(I)** Quantification of Lamin B1 expression by IF staining. A significant decrease in Lamin B1 expression was observed in low passages of Midage 1, Midage 2 and in all high passages [*n* = 4 (each n tested >15 cells), mean ± SEM, *p < 0,05, **p < 0.001, ***p < 0.0001, one-way ANOVA] **(J)** Calculated AgeScore. The AgeScore increases in cells with replicative senescence.

**FIGURE 5:**
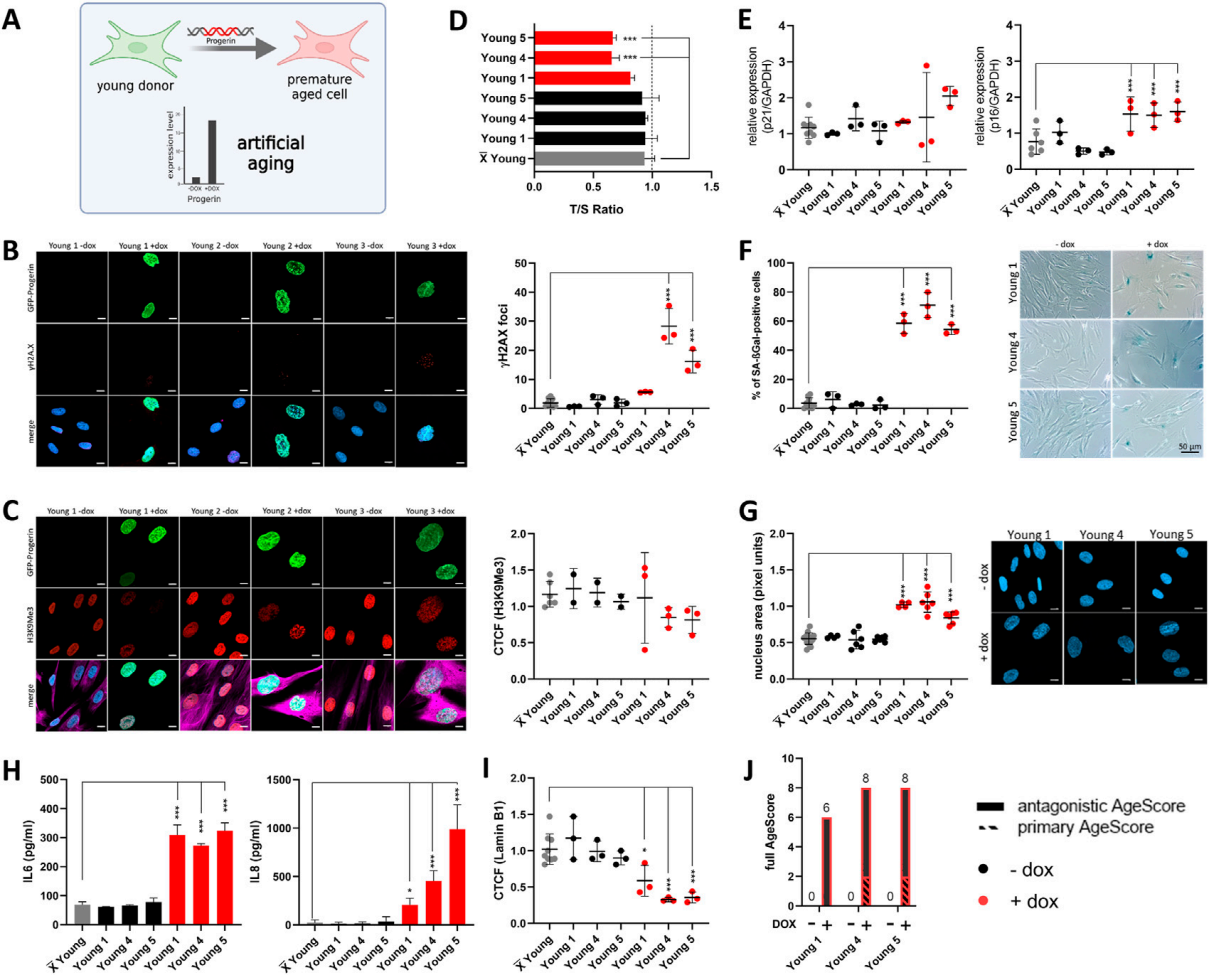
Examination of the AgeScore in premature aged human fibroblasts of young donors. Data for prematurely aged fibroblasts (induced with Doxycyclin) are marked in red. **(A)** For validation of the proposed AgeScore, young donor fibroblasts were premature aged by dox-inducible Progerin overexpression. **(B)** Representative IF images of γH2A.X and GFP-Progerin signal in absence and presence of doxycycline (1 μg/mL) after 4 days. Amount of γH2A.X foci per cell increased in cell lines with GFP-Progerin expression (Young 4, Young 5) in comparison to non-induced fibroblasts. [*n* = 3 (each n tested >15 cells), mean ± SEM,*p < 0.05, **p < 0.001, ***p < 0.0001, one-way ANOVA, scale bar = 10 µM] **(C)** Representative IF images of H3K9Me3 and GFP-Progerin signal in absence and presence of doxycycline (1 μg/mL) after 4 days (merge includes magenta = Vimentin). CTCF displayed no change in prematurely aged young donor of H3K9Me3 [*n* = 3 (each n tested >15 cells), mean ± SEM,*p < 0.05, **p < 0,001, ***p < 0.0001, one-way ANOVA, scale bar = 10 µM] **(D–I)** X̅ = Mean value of all young donors. **(D)** Telomere length was measured by MM-qPCR in comparison to a standard of healthy mixed aged individuals. In comparison to non-induced cells, Young 4 and Young 5 with progerin expression displayed a shorting of telomere length. [*n* = 3, mean ± SEM, *p < 0,05, **p < 0.001, ***p < 0.0001, one-way ANOVA] **(E)** RNA expression levels by qRT-PCR of p21 and p16 was measured to investigate activation of cell cycle arrest in human fibroblasts. Young 1, Young 4 and Young 5 with progerin expression displayed a raise of relative RNA expression level of p16 in comparison to non-induced controls. [*n* = 3, mean ± SEM *p < 0.05, **p < 0.001, ***p < 0.0001, one-way ANOVA] **(F)** Representative IF images and quantification of SA-βGAL positive cells by X-Gal staining. All three young donor cell lines with induced GFP-Progerin expression showed an increase in amount of positive stained SA-βGAL cells. [*n* = 3 (each n tested >15 cells), mean ± SEM, *p < 0.05, **p < 0.001, ***p < 0.0001, one-way ANOVA] **(G)** Representative IF images and quantification of nucleus are (pixel units) in prematurely aged human fibroblast. Nucleus area was significantly increased in all three young donor fibroblasts with induced GFP-Progerin. [*n* = 6 (each n tested >15 cells), mean ± SEM *p < 0.05, **p < 0,001, ***p < 0.0001, one-way ANOVA, scale bar: 10 µM] **(H)** Determination of cytokine secretion (IL-6, IL-8) in conditioned medium of human fibroblasts with GFP-Progerin expression in comparison to non-induced cells by ELISA. All prematurely aged fibroblasts showed a significant increase in IL-6 and IL-8 concentrations in comparison to grouped controls. [*n* = 3-4, mean ± SEM, *p < 0,05, **p < 0.001, ***p < 0.0001, one-way ANOVA] **(I)** Representative IF images and quantification of Lamin B1 IF staining. A significant decrease in Lamin B1 expression was observed in all three prematurely aged human fibroblasts [*n* = 3 (each n tested >15 cells), mean ± SEM, *p < 0.05, **p < 0.001, ***p < 0,0001, one-way ANOVA] **(J)** Calculated AgeScore for prematurely aged fibroblasts of young donors in comparison to non-induced controls.

We next aimed to examine the external validity of our proposed age-associated marker panel in two specific scenarios of aging, namely, during replicative aging/senescence (Figure 4) and artificial aging (Figure 5) by overexpression of Progerin (Miller et al., 2013).

For replicative aging (Figure 4A) we systematically compared low (*p* = 5–15) and high passages (*p*= > 30 except Old 2–4) of young, mid-age and old age donor-derived human fibroblasts. From every age group we choose two different fibroblast lines that display sufficient growth to enable passaging until high passage.

First, we investigated primary age markers. All fibroblasts in high passages (^high^) displayed significant increase in DSBs measured by γH2A.X foci in comparison to the mean of all low passages (^low^) of young donor cells (Figure 4B). We always compared to the mean of the controls and did not use a paired *t*-test to the respective low passage cell line to depict not-isogenic conditions. Expression of H3K9Me3 was reduced in Young 4^high^, Midage 1^high^, Midage 2^high^ and Old 3^high^ in comparison to grouped Young^low^ (Figure 4C). Shortening of telomeres was seen in Young 1^high^, Young 2^high^, Midage 3^high^ and Old 3^high^ compared to the mean of all Young^low^ (Figure 4D). Nucleus area (Figure 4E) and amount of positive SA-βGal stained cells (Figure 4H) was significantly increased in all cells in high passages. Next, we checked antagonistic age markers. We detected an upregulation of cell cycle arrest markers p21 and p16 in Young 1^high^, Midage 1^high^, Old 1^high^ and Old 3^high^ (Figure 4F). Furthermore, SASP activation tested by release of IL-6 and IL-8 in the cell culture medium was found in all fibroblasts with high passages in comparison to the mean of all Young^low^ (Figure 4G). Moreover, high passages of all fibroblasts displayed a decreased expression of Lamin B1 (Figure 4I). In summary, all high passaged cells homogenously display the various age markers and showed a high full AgeScore (Figure 4J).

For artificial aging, we used a doxycycline-inducible GFP-Progerin expression system (Kubben et al., 2016) in young donor cells as a model system for artificial aging. This allowed us to prematurely age the cells in a timely well-defined manner. Progerin expression indeed induced a homogenous and very rapid expression of a multitude of age markers. Four days after GFP-Progerin induction with doxycycline (1 μg/mL), prematurely aged human fibroblasts showed formation of age-related nuclear envelope phenotypes paralleled by the expression of various primary age markers. We always compared to the mean of the controls and not paired *t*-test to the respective low passage cell line to not cause a bias due to isogenic genetic background. All fibroblasts from young donors with GFP-Progerin expression displayed a significant increase of γH2A.X foci in comparison to non-induced fibroblasts (Figure 5B) whereas there were no changes in histone modification (Figure 5C). In comparison to non-induced cells, Young 4 and Young 5 with GFP-Progerin expression showed a shortening of telomere length (Figure 5D). Subsequently, we investigated the antagonistic age markers in prematurely aged fibroblasts from young donors. We observed significant increase in RNA expression of p16 in all fibroblasts with GFP-Progerin expression (Figure 5E). All prematurely aged fibroblasts showed significant increases in SA-βGal positive cell numbers (Figure 5F), nucleus area (Figure 5G) and cytokine release (Figure 5H) in comparison to non-induced cells. Furthermore, Lamin B1 expression was significantly decreased in all prematurely aged fibroblasts (Figure 5I). Generally, the AgeScore showed an increase under GFP-Progerin expression in young donor fibroblasts (Figure 5J). Progerin overexpression rapidly induced a premature aging that was in fact very similar to replicative senescence (Figure 4).

### 3.3 Fibroblasts from patients with progeria syndromes express age markers to a higher degree

**FIGURE 6:**
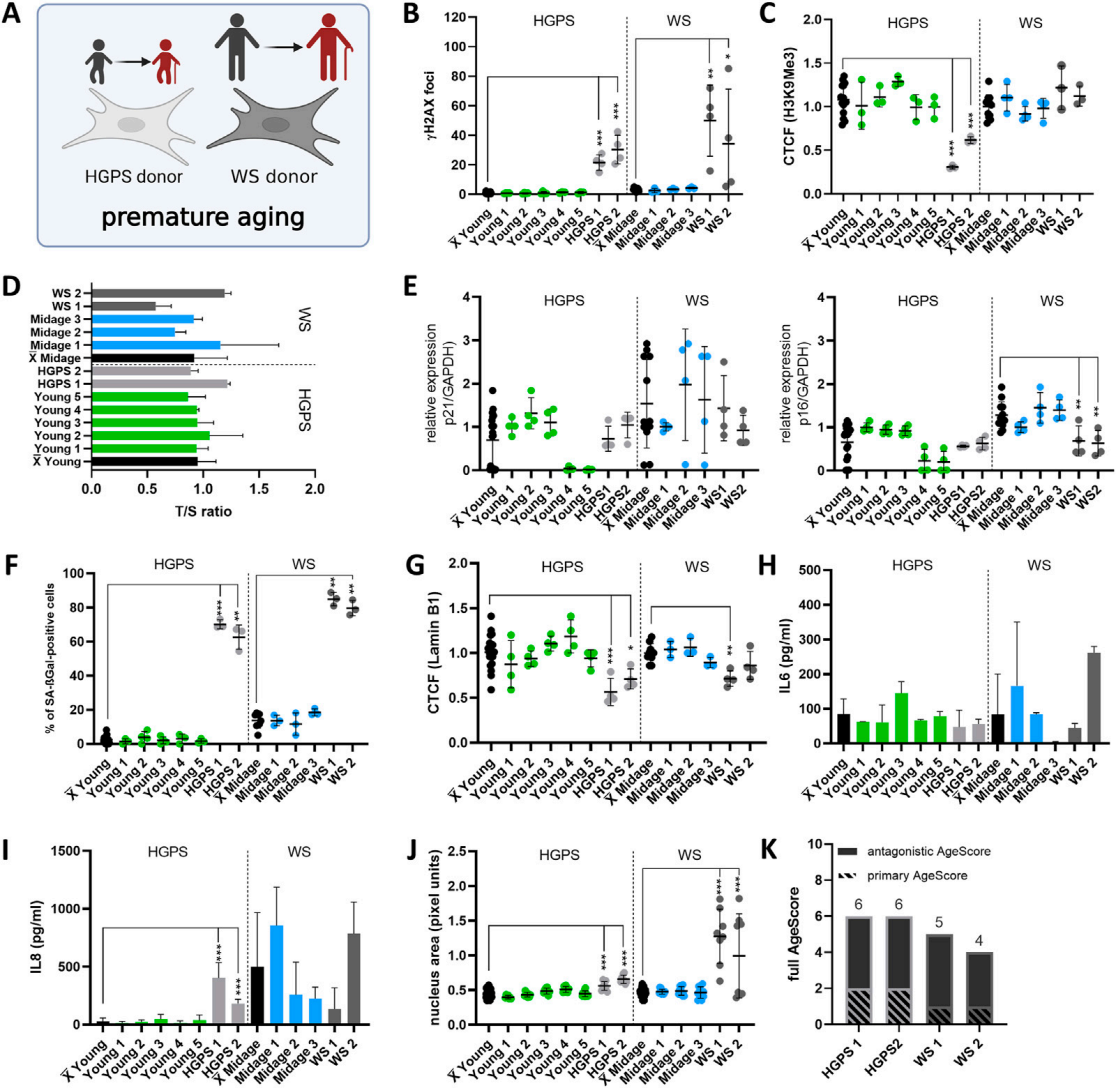
Application of the AgeScore on Progeria syndromes. **(A)** Cell Model for validation of proposed AgeScore in fibroblasts of donors with Progeria Syndrome in comparison to young/mid-age donors. **(B)** Amount of γH2A.X foci per cell examined by IF stainings to investigate DSBs. There were an increase in γH2A.X foci in all cells of the premature aging syndromes. [*n* = 3–4 (each n tested >15 cells), mean ± SEM,*p < 0,05, **p < 0.001, ***p < 0,0001, one-way ANOVA] **(C)** CTCF of H3K9Me3 in human fibroblasts of Progeria patients. There was a decrease in H3K9Me3 expression in donor fibroblasts of HGPS patients (HGPS 1 and HGPS 2). [*n* = 3–4 (each n tested >15 cells), mean ± SEM, *p < 0,05, **p < 0.001, ***p < 0.0001, one-way ANOVA] **(D)** Telomere length were measured by MM-qPCR in comparison to a standard of healthy mixed aged individuals. There were no change in telomere length of human fibroblasts of progeria syndrome patients in comparison to age-matched controls. [*n* = 3, mean ± SEM, *p < 0.05, **p < 0.001, ***p < 0.0001, one-way ANOVA] **(E)** RNA expression levels measured by qRT-PCR of p21 and p16. Activation of cell cycle arrest were detected in WS by upregulated RNA expression levels of p16. [*n* = 3-4, mean ± SEM *p < 0.05, **p < 0.001, ***p < 0.0001, one-way ANOVA] **(F)** Quantification of SA-βGAL positive cells by X-Gal staining. Donor fibroblasts from premature aging syndromes showed a significant rise of SA- βGAL positive cells. [*n* = 3–4 (each n tested >15 cells), mean ± SEM, *p < 0.05, **p < 0.001, ***p < 0.0001, one-way ANOVA] **(G)** CTCF of Lamin B1 expression. A significant decrease in Lamin B1 expression was observed in HGPS1, HGPS 2 and WS1. [*n* = 4 (each n tested >15 cells), mean ± SEM, *p < 0,05, **p < 0,001, ***p < 0.0001, one-way ANOVA] **(H,I)** Determination of cytokine secretion (IL-6, IL-8) in conditioned medium of human fibroblasts by ELISA. From studied progeria donor fibroblasts, HGPS1 and HGPS 2 displayed an increase in IL-8 concentration in comparison to the mean of age-matched controls. [*n* = 3-4, mean ± SEM, *p < 0.05, **p < 0.001, ***p < 0.0001, one-way ANOVA] **(J)** Change in nucleus morphology shown as nucleus area (pixel units). Nucleus area was significantly increased in all cells of progeria syndrome patients in comparison to age-matched controls. [*n* = 6–8 (each n tested >15 cells), mean ± SEM *p < 0.05, **p < 0.001, ***p < 0.0001, one-way ANOVA] **(K)** Calculated AgeScore of tested human fibroblasts. The AgeScores of progeria syndrome patients (HGPS 1 and 2, WS 1 and 2) are higher in comparison to the age-matched controls.

Finally, we wanted to address the question how the different aging markers behave in cell culture models of premature aging diseases. For this we studied low passage fibroblasts from patients suffering from the Progeria syndromes HGPS and WS in comparison to age matched controls, respectively (Figure 6A). Controls for HGPS were thus significantly younger than controls for WS.

The growth rates of progeria fibroblasts were investigated by PDL under stable conditions (37°C, 5% CO_2_) (Supplementary Figure S2). Fibroblasts from donors of HGPS patients showed no changes in PDL, whereas WS patient’s fibroblasts displayed slower growth compared with the mean of age-matched controls. Next, we investigated primary age markers. In progeria syndrome’s fibroblasts, we determined an increase in γH2A.X foci in all tested cells (HGPS1, HGPS 2, WS 1, and WS 2) in comparison to the mean of all age-matched controls (Figure 6B). Furthermore, we observed a decrease in H3K9Me3 expression in donor fibroblasts of HGPS patients (HGPS 1 and HGPS 2). There were no changes in fibroblasts from WS patients (Figure 6C). In comparison to grouped age-matched controls, fibroblasts of progeria patients displayed no changes in telomere length (Figure 6D).

Secondly, we checked antagonistic age markers. Activation of cell cycle arrest was detected in WS by upregulated RNA expression levels of p16 (Figure 6E). Furthermore, in all fibroblasts from progeria patients we observed a rise of SA-βGAL positive cells (Figure 6F) and a significant decrease in Lamin B1 expression in HGPS 1, HGPS 2 and WS 1 (Figure 6G). In fibroblasts of HGPS patients we observed a significant increase in concentration of IL-8 in conditioned media. There were no changes in the levels of SASP marker IL-6 or IL-8 in WS fibroblasts (Figures 6H, I). All progeria fibroblasts displayed an increase in nucleus area (Figure 6J). The AgeScores of human fibroblasts of progeria patients (HGPS 1 and 2, WS 1 and 2) were thus higher in comparison to age-matched controls and similar to those of old donor fibroblasts (Figure 6K).

**FIGURE 7:**
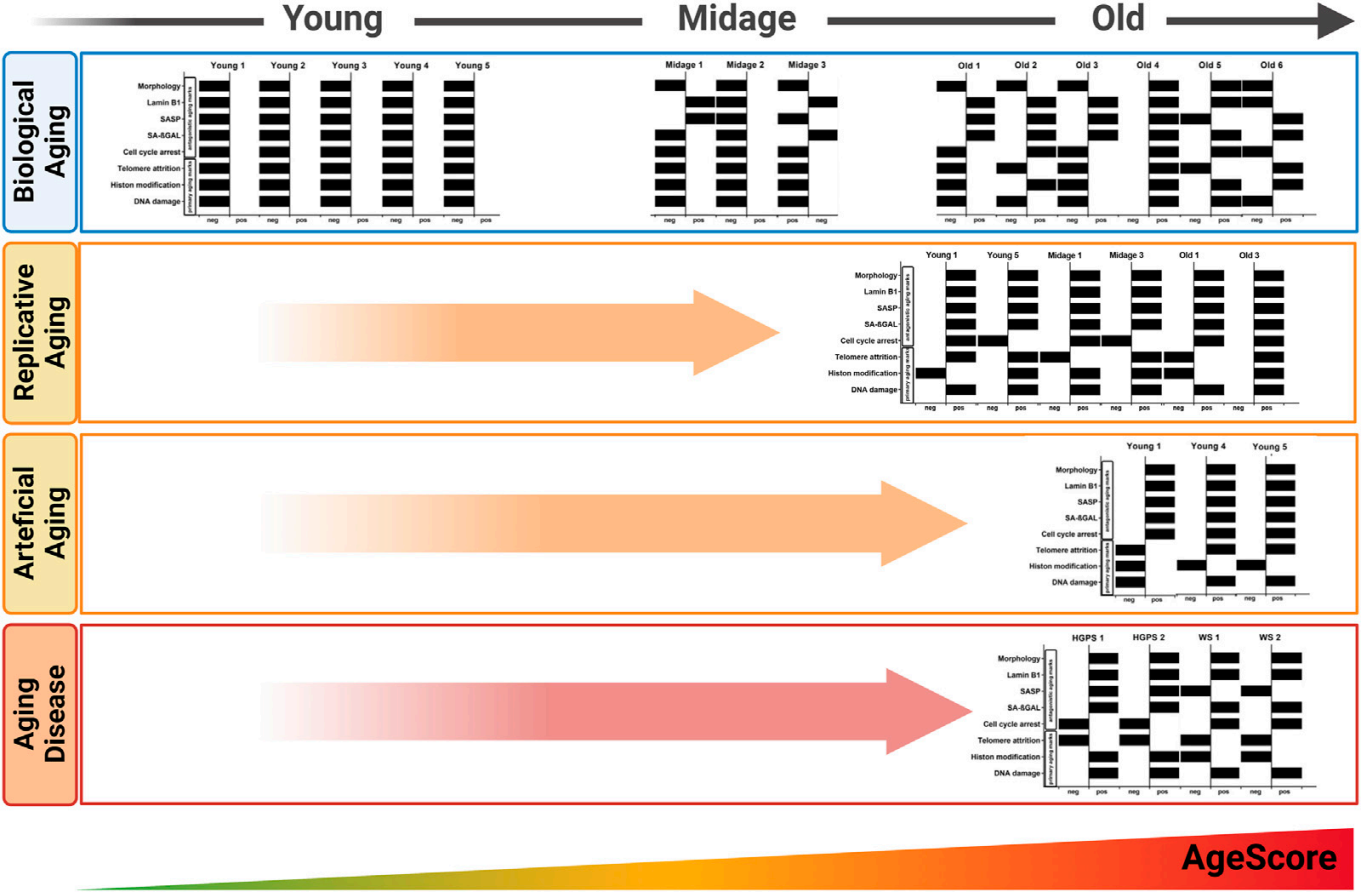
Pattern of the AgeScore. The pattern of the AgeScore between young and old donors (*in vivo* aging) increasingly shifts to the positive side depending on the age of the donor. The pattern of *in vivo* aging is similar to that of replicative and artificial aging. Here, a very strong shift to the positive test side occurs. The pattern of the *in vivo* aging process differs from the pattern of the progeria diseases, but a strong change and an increase of the AgeScore can be observed.

Interestingly, the pattern of individual markers within the AgeScore was different between HGPS cells and the cells of artificial aging, which were aged by overexpression of Progerin (Figure 7). Furthermore, the pattern of individual markers within the AgeScore were very similar between the different donors of the respective progeria syndrome, however did also differ between HGPS and WS (Figure 7). A summary of age marker expression by individual cell lines and aging conditions is presented in Table 2.

**TABLE 2:** Frequency of age marker expression per cell line investigated. Depicted is the frequency of the investigated primary and antagonistic age marker expression in the respective condition. Depicted are amount of positively tested cell lines and in brackets the total number of investigated cell lines per marker conditions, respectively.

	Donor age			Replicative aging	Artifical aging	Progeria syndromes	Frequency (total)
	Young	Midage	Old				
Primary AgeScore							
DNA damage	0 (5)	0 (3)	2 (6)	6 (6)	2 (3)	4 (4)	14 (27)
Telomere attrition	0 (5)	0 (3)	2 (6)	4 (6)	2 (3)	0 (4)	8 (27)
Histon modification	0 (5)	0 (3)	4 (6)	5 (6)	0 (3)	2 (4)	11 (27)
Antagonistic AgeScore							
SA-β-Gal	0 (5)	1 (3)	6 (6)	6 (6)	3 (3)	4 (4)	20 (27)
SASP	0 (5)	1 (3)	5 (6)	6 (6)	3 (3)	2 (4)	17 (27)
Cell cycle arrest	0 (5)	0 (3)	2 (6)	4 (6)	3 (3)	2 (4)	11 (27)
Morphology	0 (5)	0 (3)	2 (6)	6 (6)	3 (3)	4 (4)	15 (27)
LMNB1	0 (5)	2 (3)	5 (6)	6 (6)	3 (3)	3 (4)	19 (27)

## 4 Discussion

Despite the existence of a variety of putative age markers, comparison of different studies on aging cells is hampered by the fact that often only single markers are used (López-otín et al., 2013; Hartmann et al., 2021). Here, we systematically investigated a set of established age markers in diverse cellular aging conditions. Doing so, we propose a panel of various commonly used age markers that together seem to measure the effect of aging processes on cells at least as good as single markers but additionally giving insights in the complexity of the aging process (Figure 7; Table 2). Additionally, the panel might be helpful to compensate for bias due to genetic background differences.

Cell models of disease and/or aging are of even greater importance since the discovery of iPSC and other “induced” cell technologies. While these technologies enable to investigate patient-derived cell models of previously unavailable origin (e.g., neurons), they significantly alter also general aspects of the cell including their biological age. Different age scores/clocks have thus been proposed in the last years which are estimating biological age with various degrees of accuracy (Lu et al., 2019; Tarkhov, Denisov, and Fedichev, 2022). It is well known, that aging leads to epigenetic changes in DNA methylation, through several distinct and overlapping age-associated mechanisms (Hannum et al., 2013; López-otín et al., 2013). Also, many DNA methylation clocks, which allow the indirect inference of biological age from age-specific DNA methylation patterns, have been recently established and enable the estimation of biological age of organisms in large tissue samples followed by multivariate machine learning models (Hannum et al., 2013; Horvath, 2013; Robinson et al., 2020; Van Den Akker et al., 2020; Hwangbo et al., 2021). Nevertheless, for the use in standard cell culture these investigations are often expensive and need specific equipment not necessarily available.

All cells characterized in this study showed quite diverse age marker expression, visible in the here proposed age-associated marker panel (Figure 7; Table 2). This demonstrates two concepts: On the one hand it potentially depicts the individuality of the aging process *per se*, which is based, amongst others, on the genetic heterogeneity of donors (Schneider and Mitsui, 1976). On the other hand, it also displays the heterogeneity of individual marker expression and the flaws when only looking at individual markers. The age-associated marker panel from donors with different ages showed an increase with chronological age, whereby old donor cells displayed a higher heterogeneity. For example, fibroblasts from Old 4 had the highest score of 9, but the donor of Old 4 was younger than Old 6 with a score of 4. Nevertheless, in comparison with young donors (all scoring 0) and mid-age donors (scoring 0–2), it becomes clear that scores increased markedly with age. Furthermore, replicative aging induced highest scores, higher than donor age itself. Thus, the score might be most sensitive for the detection of replicative senescence, or the markers chosen might be overrepresented in replicative senescence. Of note, the score still increased with replicative aging even in case of being already increased due to donor age. In addition, initial studies on premature aging diseases (Figure 6) pointed towards increased scores in both. One might discuss to introduce an additional weighting system for the AgeScore, e.g., by taking into account the frequency of the respective marker expression in the different age/aging conditions (Table 2). Such a weighting might, however, not generalize to other cell lines or “induced” cell models, thus requiring further refinement and validation.

Interestingly, “antagonistic age markers” appeared earlier than “primary age markers”. Antagonistic age markers were always expressed in replicative/artificial aging and were already present in mid-age donors. While these were also positive without primary age markers being increased, this was never the case *vice versa*. We cannot, however, rule out that this might be due to detection limits of the respective markers.

Heterogeneity of the different marker expression was highest when investigating donor age dependency. This heterogeneity might be due to differences in the genetic background of the different donors but also due to differences in the biological age (meaning their health state at biopsy) of the respective donors themselves. Ideally this would mean that the biological age of the respective donor has also been estimated using an age score. In practice, however, this biological age of the donors is often not available. This heterogeneity was less pronounced in case of replicative senescence and induced accelerated aging. In order to minimize this heterogeneity in the latter, we compared the replicative senescence and the progerin overexpression induced aging not to the respective donor cell (=isogenic background) but to the mean of all control conditions. Nevertheless, replicative and progerin induced senescence experimental settings might be more defined than aging in an individual prior to cell isolation with or without premature aging disease.

In addition, we examined the pattern of the individual markers within the age-associated marker panel of the cells and in the conditions studied (Figure 7). This additionally underpins the observation that aging processes are diverse, and might also account for deviations between the plurality of AgeClocks described so far. When looking at the panel, we saw a clear change in the pattern of these different markers according to donor age, but also marked differences between different donors of similar age. Even though all markers chosen were reported to be age associated or increase with aging, we only rarely found all of them increased together. Specific markers such as SA-β-Galactosidase were associated clearly with aging and positive cells increase in nearly all aging conditions (Figure 7; Table 2), but they still had minor problems e.g., to label clearly all mid-age donors (Figure 2). Interestingly, another age scoring system—so called CultureAge—did even report a lack of correlation of SA-β-Galactosidase with CultureAge (Minteer et al., 2022). Other age markers like p21 displayed an increase only in few of the tested cells. Replicative senescence and artificial aging induced a stronger and especially more homogenous change of the overall marker pattern. While total score of the age marker panel was even higher in Werner syndrome fibroblasts, the patterns of HGPS and WS were remarkable different.

A limitation of our study is the exclusive use of human dermal fibroblasts. It is well accepted that cellular aging differs across tissues *in vivo* and *in vitro*. In addition, the expression of specific markers, e.g., p16 and p21, is tissue dependent. Consequently, the AgeScore might be affected as well by the cell origin. Furthermore, some markers investigated only seldomly became positive in aged conditions despite having been described as established age markers (Table 2). Thus future studies are warranted to both investigate the value of the AgeScore and its marker panel in cells from diverse tissue/germ layer origin including (re-)programming conditions, and to eventually reduce the AgeScore to a panel of markers, which all become positive in aged conditions, irrespective of tissue origin.

In summary, we here propose the use of an age-associated marker panel, which considers key age markers for application in *in vitro* cell culture and demonstrated its validity in estimating donor age, replicative and artificial aging as well as premature aging. This AgeScore can be easily investigated in standard cell culture laboratories yielding both an individual value but also marker patterns that allow good comparability of the diversity of the aging processes. Further studies are needed to further validate the use of the AgeScore proposed here, also for other *in vitro* and *in vivo* applications.

## Data Availability

The raw data supporting the conclusion of this article will be made available by the authors, without undue reservation.
