# Supplementary material for: Systematic estimation of biological age of in vitro cell culture systems by an age-associated marker panel
Source: Front Aging. 2023 Feb 15;4:1129107. doi: 10.3389/fragi.2023.1129107 (PMC9975507; doi:10.3389/fragi.2023.1129107)
Supplement: Supplementary file 1 [file DataSheet1.docx]

Supplementary Material

Systematic estimation of biological age of *in vitro* cell culture systems by an age-associated marker panel

**Hartmann, Christiane^1^; Herling, Luise^1^; Hartmann, Alexander^3^; Köckritz, Verena^1^, Fuellen, Georg ^4,5^, Walter, Michael^3,5^; Hermann, Andreas*^1,2,5^**

^1^ Translational Neurodegeneration Section “Albrecht-Kossel”, Department of Neurology, University Medical Center Rostock, Rostock, Germany

^2^ Deutsches Zentrum für Neurodegenerative Erkrankungen (DZNE) Rostock/Greifswald, Rostock, Germany

^3^ Institute of Clinical Chemistry and Laboratory Medicine, University Medical Center Rostock, Rostock, Germany

^4^ Institute for Biostatistics and Informatics in Medicine and Ageing Research, Rostock University Medical Center, Rostock, Germany

^5^ Center for Transdisciplinary Neurosciences Rostock (CTNR), University Medical Center Rostock, Rostock, Germany

***Correspondence:** Andreas Hermann, Translational Neurodegeneration Section “Albrecht-Kossel”, Department of Neurology, University Medical Center Rostock, Rostock, Germany, [Andreas.hermann@med.uni-rostock.de](mailto:Andreas.hermann@med.uni-rostock.de)

# Supplementary Figures and Tables

## Supplementary Figures

**Supplementary Figure 1: Pearson’s Correlation of AgeScore and used age markers.** **(A - J)** Calculation of Pearson’s Correlation of each used age marker included in the here proposed AgeScore. The results display a very high correlation for the markers H3K9Me3 (B), p21 (D), nucleus size (J) and SA-ß-Gal and a high correlation for Lamin B1 (G) and H2AX (A). However, there is a moderate correlation for the telomere length (C) and a low correlation for p16 (E), IL6 (H), and IL8 (I).


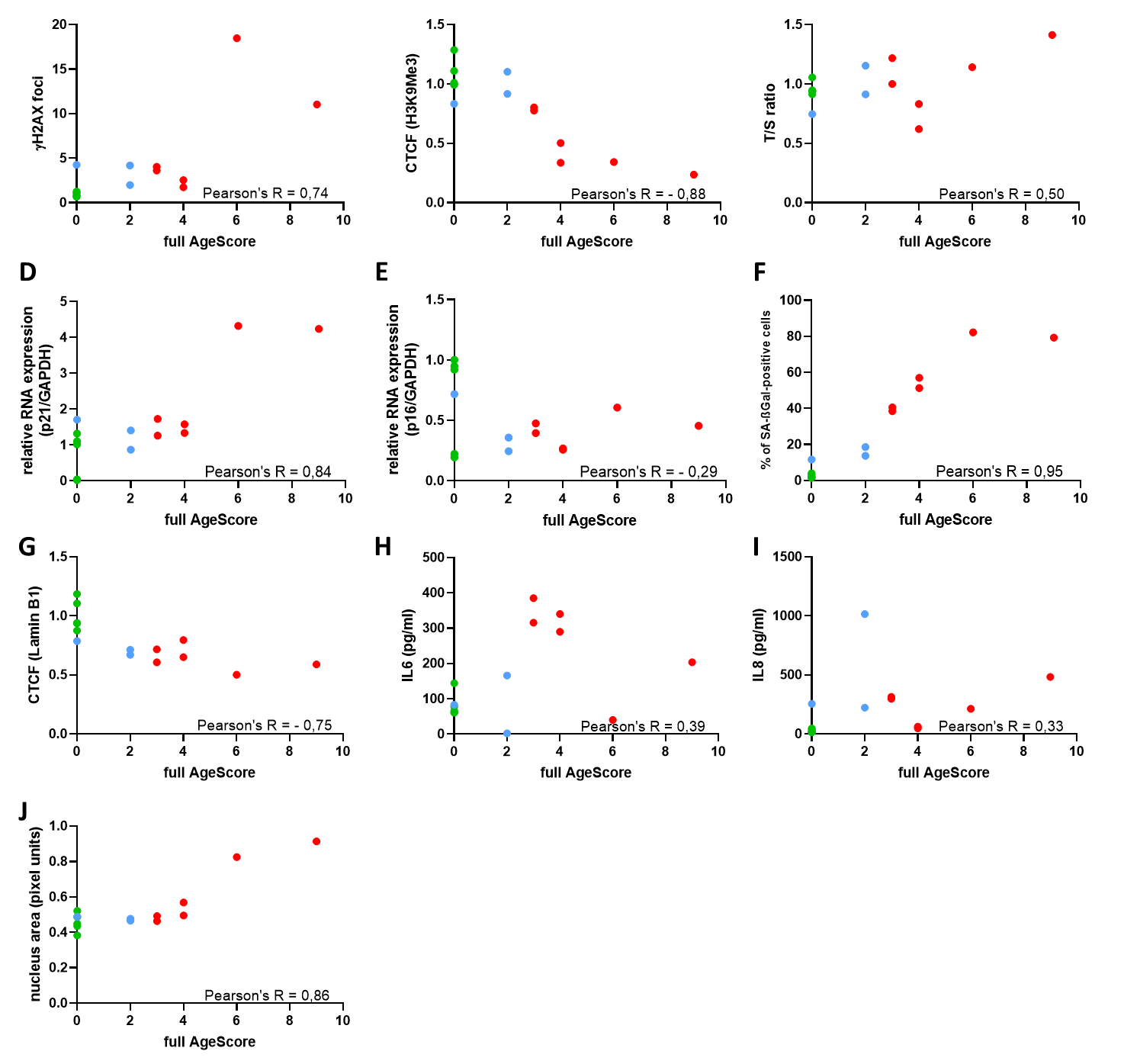

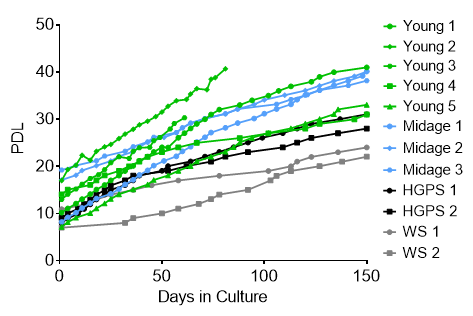


**Supplementary Figure 2: Growth curve of Progeria Syndrome fibroblasts.** The replicative potential of human fibroblasts used for examining the AgeScore. Cells were cultured under stable conditions (37°C, 5% CO2) in DMEM medium (with 15% FBS, 1% Pen-Strep). PDL was observed over 150 days. Cells from donors of HGPS patients displayed no changes in PDL, whereas WS patient’s fibroblasts showed slower growth compared with the mean of age-matched controls.

## Supplementary Tables

**Table 1: Age markers and their detection methods**

| Hallmark Category | Aging Hallmark | Example of age marker | Methods | Publications |
| --- | --- | --- | --- | --- |
| Primary Hallmarks (causes of damage) | Genomic instability | Decrease of Lamin B1 expression | WB,  IF | (A. S. Wang et al. 2017), (Dreesen, Ong, et al. 2013; Dreesen, Chojnowski, et al. 2013),(Freund et al. 2012),(Shimi et al. 2011) |
|  |  | DNA damage accumulation | WB, IF | (Schumacher et al. 2021),(Mah, El-Osta, and Karagiannis 2010),(Sedelnikova et al. 2008),(Sedelnikova et al. 2004)(Redon et al. 2002) |
|  | Telomere Attrition | Telomere length | qPCR, TRF, TCA | (Liu et al. 2019);(Armanios et al. 2009);(Bodnar et al. 2016);(Allsopp et al. 1992) |
|  | Epigenetic Alterations | Histone Modification | WB, IF | (Zhang et al. 2016);(McCauley and Dang 2014);(Greer et al. 2010);(Siebold et al. 2010); |
|  |  | DNA Methylation | WB, IF | (Salameh, Bejaoui, and El Hajj 2020);(Sturm et al. 2019);(Horvath 2013);(Osorio et al. 2010);(Shumaker et al. 2006) |
|  |  | Transcriptional alteration | RNA-Sequencing | (Aramillo Irizar et al. 2018);(Harries et al. 2011);(Bahar et al. 2006)(Marthandan et al. 2016) |
|  | Loss of Proteostasis | Expression of heat shock proteins | WB, IF | (Koga, Kaushik, and Cuervo 2011);(Terry et al. 2006);(Wilhelmus et al. 2006);(Marini et al. 2004);(Cavinato et al. 2017) |
| Antagonistic Hallmarks (Responses to damage) | Deregulated Nutrient Sensing | Expression of proteins of AKT/mTOR/FOXO-pathways | WB, IF | (Barzilai et al. 2012); (Kenyon 2010) |
|  | Mitochondrial Dysfunction | ROS production | SOD/Catalase activity assay | (Damiani et al. 2018);(Green, Galluzzi, and Kroemer 2011);(Hekimi, Lapointe, and Wen 2011);(Mesquita et al. 2010);(Doonan et al. 2008) |
|  | Cellular Senescence | SA-β-Galactosidase | SA-β-Gal assay | (Kuilman et al. 2010);(C. Wang et al. 2009);(Dimri et al. 1995) |
|  |  | SASP | qPCR,  ELISA | (Basisty et al. 2020);(Acosta et al. 2013);(Coppé et al. 2008);(Collado, Blasco, and Serrano 2007); |
|  |  | cell cycle arrest | qPCR,  WB | (Ressler et al. 2006); (Krishnamurthy et al. 2004);(Serrano et al. 1997);(Hayflick and Moorhead 1961) |
| Integrative Hallmarks (Phenotype Inducer) | Stem Cell Exhaustion | cell amount | FACS | (Rera et al. 2011);(Rossi et al. 2007);(Sharpless and DePinho 2007); |
|  | Altered Intercellular Communication | e.g inflammaging | various | (Laplante and Sabatini 2012); (Salminen, Kaarniranta, and Kauppinen 2012);(Pont et al. 2012);(Durieux, Wolff, and Dillin 2011)(Lee et al. 2021) |

Acosta, Juan Carlos, Ana Banito, Torsten Wuestefeld, Athena Georgilis, Peggy Janich, Jennifer P. Morton, Dimitris Athineos, et al. 2013. “A Complex Secretory Program Orchestrated by the Inflammasome Controls Paracrine Senescence.” *Nature Cell Biology* 15 (8): 978–90. https://doi.org/10.1038/ncb2784.

Allsopp, R. C., H. Vaziri, C. Patterson, S. Goldstein, E. V. Younglai, A. B. Futcher, C. W. Greider, and C. B. Harley. 1992. “Telomere Length Predicts Replicative Capacity of Human Fibroblasts.” *Proceedings of the National Academy of Sciences of the United States of America* 89 (21): 10114–18. https://doi.org/10.1073/pnas.89.21.10114.

Aramillo Irizar, Peer, Sascha Schäuble, Daniela Esser, Marco Groth, Christiane Frahm, Steffen Priebe, Mario Baumgart, et al. 2018. “Transcriptomic Alterations during Ageing Reflect the Shift from Cancer to Degenerative Diseases in the Elderly.” *Nature Communications* 9 (1): 1–11. https://doi.org/10.1038/s41467-017-02395-2.

Armanios, Mary, Jonathan K. Alder, Erin M. Parry, Baktiar Karim, Margaret A. Strong, and Carol W. Greider. 2009. “Short Telomeres Are Sufficient to Cause the Degenerative Defects Associated with Aging.” *American Journal of Human Genetics* 85 (6): 823–32. https://doi.org/10.1016/j.ajhg.2009.10.028.

Bahar, Rumana, Claudia H. Hartmann, Karl A. Rodriguez, Ashley D. Denny, Rita A. Busuttil, Martijn E.T. Dollé, R. Brent Calder, et al. 2006. “Increased Cell-to-Cell Variation in Gene Expression in Ageing Mouse Heart.” *Nature* 441 (7096): 1011–14. https://doi.org/10.1038/nature04844.

Barzilai, Nir, Derek M. Huffman, Radhika H. Muzumdar, and Andrzej Bartke. 2012. “The Critical Role of Metabolic Pathways in Aging.” *Diabetes* 61 (6): 1315–22. https://doi.org/10.2337/db11-1300.

Basisty, Nathan, Abhijit Kale, Ok Hee Jeon, Chisaka Kuehnemann, Therese Payne, Chirag Rao, Anja Holtz, et al. 2020. “A Proteomic Atlas of Senescence-Associated Secretomes for Aging Biomarker Development.” *PLoS Biology* 18 (1): e3000599. https://doi.org/10.1371/journal.pbio.3000599.

Bodnar, Andrea G, Michel Ouellette, Maria Frolkis, Shawn E Holt, Gregg B Morin, Calvin B Harley, Jerry W Shay, et al. 2016. “Extension of Life-Span by Introduction of Telomerase into Normal Human Cells Published by : American Association for the Advancement of Science Stable URL : Http://Www.Jstor.Org/Stable/2894563 REFERENCES Linked References Are Available on JSTOR for This A” 279 (5349): 349–52.

Cavinato, Maria, Rafal Koziel, Nikolaus Romani, Regina Weinmüllner, Brigitte Jenewein, Martin Hermann, Sandrine Dubrac, et al. 2017. “UVB-Induced Senescence of Human Dermal Fibroblasts Involves Impairment of Proteasome and Enhanced Autophagic Activity.” *Journals of Gerontology - Series A Biological Sciences and Medical Sciences* 72 (5): 632–39. https://doi.org/10.1093/gerona/glw150.

Collado, Manuel, Maria A. Blasco, and Manuel Serrano. 2007. “Cellular Senescence in Cancer and Aging.” *Cell* 130 (2): 223–33. https://doi.org/10.1016/j.cell.2007.07.003.

Coppé, Jean Philippe, Christopher K. Patil, Francis Rodier, Y. Sun, Denise P. Muñoz, Joshua Goldstein, Peter S. Nelson, Pierre Yves Desprez, and Judith Campisi. 2008. “Senescence-Associated Secretory Phenotypes Reveal Cell-Nonautonomous Functions of Oncogenic RAS and the P53 Tumor Suppressor.” *PLoS Biology* 6 (12). https://doi.org/10.1371/journal.pbio.0060301.

Damiani, Elisabetta, Francesca Brugè, Ilenia Cirilli, Fabio Marcheggiani, Fabiola Olivieri, Tatiana Armeni, Laura Cianfruglia, Angelica Giuliani, Patrick Orlando, and Luca Tiano. 2018. “Modulation of Oxidative Status by Normoxia and Hypoxia on Cultures of Human Dermal Fibroblasts: How Does It Affect Cell Aging?” *Oxidative Medicine and Cellular Longevity* 2018. https://doi.org/10.1155/2018/5469159.

Dimri, Goberdhan P., Xinhua Lee, George Basile, Meileen Acosta, Glynis Scott, Calvin Roskelley, Estela E. Medrano, et al. 1995. “A Biomarker That Identifies Senescent Human Cells in Culture and in Aging Skin in Vivo.” *Proceedings of the National Academy of Sciences of the United States of America* 92 (20): 9363–67. https://doi.org/10.1073/pnas.92.20.9363.

Doonan, Ryan, Joshua J. McElwee, Filip Matthijssens, Glenda A. Walker, Koen Houthoofd, Patricia Back, Andrea Matscheski, Jacques R. Vanfleteren, and David Gems. 2008. “Against the Oxidative Damage Theory of Aging: Superoxide Dismutases Protect against Oxidative Stress but Have Little or No Effect on Life Span in Caenorhabditis Elegans.” *Genes and Development* 22 (23): 3236–41. https://doi.org/10.1101/gad.504808.

Dreesen, Oliver, Alexandre Chojnowski, Peh Fern Ong, Tian Yun Zhao, John E. Common, Declan Lunny, E. Birgitte Lane, et al. 2013. “Lamin B1 Fluctuations Have Differential Effects on Cellular Proliferation and Senescence.” *Journal of Cell Biology* 200 (5): 605–17. https://doi.org/10.1083/jcb.201206121.

Dreesen, Oliver, Peh Fern Ong, Alexandre Chojnowski, and Alan Colman. 2013. “The Contrasting Roles of Lamin B1 in Cellular Aging and Human Disease.” *Nucleus* 4 (4): 283–90. https://doi.org/10.4161/nucl.25808.

Durieux, Jenni, Suzanne Wolff, and Andrew Dillin. 2011. “The Cell-Non-Autonomous Nature of Electron Transport Chain-Mediated Longevity.” *Cell* 144 (1): 79–91. https://doi.org/10.1016/j.cell.2010.12.016.

Freund, Adam, Remi Martin Laberge, Marco Demaria, and Judith Campisi. 2012. “Lamin B1 Loss Is a Senescence-Associated Biomarker.” *Molecular Biology of the Cell* 23 (11): 2066–75. https://doi.org/10.1091/mbc.E11-10-0884.

Green, Douglas R., Lorenzo Galluzzi, and Guido Kroemer. 2011. “Mitochondria and the Autophagy-Inflammation-Cell Death Axis in Organismal Aging.” *Science* 333 (6046): 1109–12. https://doi.org/10.1126/science.1201940.

Greer, Eric L., Travis J. Maures, Anna G. Hauswirth, Erin M. Green, Dena S. Leeman, Géraldine S. Maro, Shuo Han, Max R. Banko, Or Gozani, and Anne Brunet. 2010. “Members of the H3K4 Trimethylation Complex Regulate Lifespan in a Germline-Dependent Manner in C. Elegans.” *Nature* 466 (7304): 383–87. https://doi.org/10.1038/nature09195.

Harries, Lorna W, Dena Hernandez, William Henley, Andrew R Wood, Alice C Holly, Rachel M, Hanieh Yaghootkar, et al. 2011. “Expression and Deregulation of Alternative Splicing Aging Cell.” *Aging Cell* 10 (5): 868–78. https://doi.org/10.1111/j.1474-9726.2011.00726.x.Human.

Hayflick, L., and P. S. Moorhead. 1961. “The Serial Cultivation of Human Diploid Cell Strains.” *Experimental Cell Research* 25 (3): 585–621. https://doi.org/10.1016/0014-4827(61)90192-6.

Hekimi, Siegfried, Jérôme Lapointe, and Yang Wen. 2011. “Taking a ‘Good’ Look at Free Radicals in the Aging Process.” *Trends in Cell Biology* 21 (10): 569–76. https://doi.org/10.1016/j.tcb.2011.06.008.

Horvath, Steve. 2013. “DNA Methylation Age of Human Tissues and Cell Types.” *Genome Biology* 14 (10). https://doi.org/10.1186/gb-2013-14-10-r115.

Kenyon, Cynthia J. 2010. “The Genetics of Ageing.” *Nature* 464 (7288): 504–12. https://doi.org/10.1038/nature08980.

Koga, Hiroshi, Susmita Kaushik, and Ana Maria Cuervo. 2011. “Protein Homeostasis and Aging: The Importance of Exquisite Quality Control.” *Ageing Research Reviews* 10 (2): 205–15. https://doi.org/10.1016/j.arr.2010.02.001.

Krishnamurthy, Janakiraman, Chad Torrice, Matthew R. Ramsey, Grigoriy I. Kovalev, Khalid Al-Regaiey, Lishan Su, and Norman E. Sharpless. 2004. “Ink4a/Arf Expression Is a Biomarker of Aging.” *Journal of Clinical Investigation* 114 (9): 1299–1307. https://doi.org/10.1172/JCI22475.

Kuilman, Thomas, Chrysiis Michaloglou, Wolter J. Mooi, and Daniel S. Peeper. 2010. “The Essence of Senescence.” *Genes and Development* 24 (22): 2463–79. https://doi.org/10.1101/gad.1971610.

Laplante, Mathieu, and David M. Sabatini. 2012. “MTOR Signaling in Growth Control and Disease.” *Cell* 149 (2): 274–93. https://doi.org/10.1016/j.cell.2012.03.017.

Lee, Young In, Sooyeon Choi, Won Seok Roh, Ju Hee Lee, and Tae Gyun Kim. 2021. “Cellular Senescence and Inflammaging in the Skin Microenvironment.” *International Journal of Molecular Sciences* 22 (8). https://doi.org/10.3390/ijms22083849.

Liu, Jun, Lihui Wang, Zhiguo Wang, and Jun-Ping Liu. 2019. “Replicative and Chronological Ageing.” *Cells*, 1–10.

Mah, Li Jeen, Assam El-Osta, and Tom C. Karagiannis. 2010. “ΓH2AX as a Molecular Marker of Aging and Disease.” *Epigenetics* 5 (2): 129–36. https://doi.org/10.4161/epi.5.2.11080.

Marini, Marina, Rosa Lapalombella, Silvia Canaider, Antonio Farina, Daniela Monti, Valentina De Vescovi, Marina Morellini, et al. 2004. “Heat Shock Response by EBV-Immortalized B-Lymphocytes from Centenarians and Control Subjects: A Model to Study the Relevance of Stress Response in Longevity.” *Experimental Gerontology* 39 (1): 83–90. https://doi.org/10.1016/j.exger.2003.09.023.

Marthandan, S., M. Baumgart, S. Priebe, M. Groth, J. Schaer, C. Kaether, R. Guthke, et al. 2016. “Conserved Senescence Associated Genes and Pathways in Primary Human Fibroblasts Detected by RNA-Seq.” *PLoS ONE* 11 (5): 1–31. https://doi.org/10.1371/journal.pone.0154531.

McCauley, Brenna S., and Weiwei Dang. 2014. “Histone Methylation and Aging: Lessons Learned from Model Systems.” *Biochimica et Biophysica Acta - Gene Regulatory Mechanisms* 1839 (12): 1454–62. https://doi.org/10.1016/j.bbagrm.2014.05.008.

Mesquita, Ana, Martin Weinberger, Alexandra Silva, Belém Sampaio-Marques, Bruno Almeida, Cecília Leão, Vítor Costa, Fernando Rodrigues, William C. Burhans, and Paula Ludovico. 2010. “Caloric Restriction or Catalase Inactivation Extends Yeast Chronological Lifespan by Inducing H2O2 and Superoxide Dismutase Activity.” *Proceedings of the National Academy of Sciences of the United States of America* 107 (34): 15123–28. https://doi.org/10.1073/pnas.1004432107.

Osorio, Fernando G., Ignacio Varela, Ester Lara, Xose S. Puente, Jesús Espada, Raffaella Santoro, José M.P. Freije, Mario F. Fraga, and Carlos López-Otín. 2010. “Nuclear Envelope Alterations Generate an Aging-like Epigenetic Pattern in Mice Deficient in Zmpste24 Metalloprotease.” *Aging Cell* 9 (6): 947–57. https://doi.org/10.1111/j.1474-9726.2010.00621.x.

Pont, Adam R., Navid Sadri, Susan J. Hsiao, Susan Smith, and Robert J. Schneider. 2012. “MRNA Decay Factor AUF1 Maintains Normal Aging, Telomere Maintenance, and Suppression of Senescence by Activation of Telomerase Transcription.” *Molecular Cell* 47 (1): 5–15. https://doi.org/10.1016/j.molcel.2012.04.019.

Redon, Christophe, Duane Pilch, Emmy Rogakou, Olga Sedelnikova, Kenneth Newrock, and William Bonner. 2002. “Histone H2A Variants H2AX and H2AZ.” *Current Opinion in Genetics and Development* 12 (2): 162–69. https://doi.org/10.1016/S0959-437X(02)00282-4.

Rera, Michael, Sepehr Bahadorani, Jaehyoung Cho, Christopher L. Koehler, Matthew Ulgherait, Jae H. Hur, William S. Ansari, Thomas Lo, D. Leanne Jones, and David W. Walker. 2011. “Modulation of Longevity and Tissue Homeostasis by the Drosophila PGC-1 Homolog.” *Cell Metabolism* 14 (5): 623–34. https://doi.org/10.1016/j.cmet.2011.09.013.

Ressler, Sigrun, Jirina Bartkova, Harald Niederegger, Jiri Bartek, Karin Scharffetter-Kochanek, Pidder Jansen-Dürr, and Meinhard Wlaschek. 2006. “P16INK4A Is a Robust in Vivo Biomarker of Cellular Aging in Human Skin.” *Aging Cell* 5 (5): 379–89. https://doi.org/10.1111/j.1474-9726.2006.00231.x.

Rossi, Derrick J., David Bryder, Jun Seita, Andre Nussenzweig, Jan Hoeijmakers, and Irving L. Weissman. 2007. “Deficiencies in DNA Damage Repair Limit the Function of Haematopoietic Stem Cells with Age.” *Nature* 447 (7145): 725–29. https://doi.org/10.1038/nature05862.

Salameh, Yasmeen, Yosra Bejaoui, and Nady El Hajj. 2020. “DNA Methylation Biomarkers in Aging and Age-Related Diseases.” *Frontiers in Genetics* 11 (March): 1–11. https://doi.org/10.3389/fgene.2020.00171.

Salminen, Antero, Kai Kaarniranta, and Anu Kauppinen. 2012. “Inflammaging: Disturbed Interplay between Autophagy and Inflammasomes.” *Aging* 4 (3): 166–75. https://doi.org/10.18632/aging.100444.

Schumacher, Björn, Joris Pothof, Jan Vijg, and Jan H.J. Hoeijmakers. 2021. “The Central Role of DNA Damage in the Ageing Process.” *Nature* 592 (7856): 695–703. https://doi.org/10.1038/s41586-021-03307-7.

Sedelnikova, Olga A., Izumi Horikawa, Christophe Redon, Asako Nakamura, Drazen B. Zimonjic, Nicholas C. Popescu, and William M. Bonner. 2008. “Delayed Kinetics of DNA Double-Strand Break Processing in Normal and Pathological Aging.” *Aging Cell* 7 (1): 89–100. https://doi.org/10.1111/j.1474-9726.2007.00354.x.

Sedelnikova, Olga A., Izumi Horikawa, Drazen B. Zimonjic, Nicholas C. Popescu, William M. Bonner, and J. Carl Barrett. 2004. “Senescing Human Cells and Ageing Mice Accumulate DNA Lesions with Unrepairable Double-Strand Breaks.” *Nature Cell Biology* 6 (2): 168–70. https://doi.org/10.1038/ncb1095.

Serrano, Manuel, Athena W. Lin, Mila E. McCurrach, David Beach, and Scott W. Lowe. 1997. “Oncogenic Ras Provokes Premature Cell Senescence Associated with Accumulation of P53 and P16(INK4a).” *Cell* 88 (5): 593–602. https://doi.org/10.1016/S0092-8674(00)81902-9.

Sharpless, Norman E., and Ronald A. DePinho. 2007. “How Stem Cells Age and Why This Makes Us Grow Old.” *Nature Reviews Molecular Cell Biology* 8 (9): 703–13. https://doi.org/10.1038/nrm2241.

Shimi, Takeshi, Veronika Butin-Israeli, Stephen A. Adam, Robert B. Hamanaka, Anne E. Goldman, Catherine A. Lucas, Dale K. Shumaker, Steven T. Kosak, Navdeep S. Chandel, and Robert D. Goldman. 2011. “The Role of Nuclear Lamin B1 in Cell Proliferation and Senescence.” *Genes and Development* 25 (24): 2579–93. https://doi.org/10.1101/gad.179515.111.

Shumaker, Dale K., Thomas Dechat, Alexander Kohlmaier, Stephen A. Adam, Matthew R. Bozovsky, Michael R. Erdos, Maria Eriksson, et al. 2006. “Mutant Nuclear Lamin A Leads to Progressive Alterations of Epigenetic Control in Premature Aging.” *Proceedings of the National Academy of Sciences of the United States of America* 103 (23): 8703–8. https://doi.org/10.1073/pnas.0602569103.

Siebold, Alex P., Rakhee Banerjee, Feng Tie, Daniel L. Kiss, Jacob Moskowitz, and Peter J. Harte. 2010. “Polycomb Repressive Complex 2 and Trithorax Modulate Drosophila Longevity and Stress Resistance.” *Proceedings of the National Academy of Sciences of the United States of America* 107 (1): 169–74. https://doi.org/10.1073/pnas.0907739107.

Sturm, Gabriel, Andres Cardenas, Marie Abèle Bind, Steve Horvath, Shuang Wang, Yunzhang Wang, Sara Hägg, Michio Hirano, and Martin Picard. 2019. “Human Aging DNA Methylation Signatures Are Conserved but Accelerated in Cultured Fibroblasts.” *Epigenetics* 14 (10): 961–76. https://doi.org/10.1080/15592294.2019.1626651.

Terry, Dellara F., Diego F. Wyszynski, Vikki G. Nolan, Gil Atzmon, Emily A. Schoenhofen, Jae Mi Y. Pennington, Stacy L. Andersen, et al. 2006. “Serum Heat Shock Protein 70 Level as a Biomarker of Exceptional Longevity.” *Mechanisms of Ageing and Development* 127 (11): 862–68. https://doi.org/10.1016/j.mad.2006.08.007.

Wang, Audrey Shimei, Peh Fern Ong, Alexandre Chojnowski, Carlos Clavel, and Oliver Dreesen. 2017. “Loss of Lamin B1 Is a Biomarker to Quantify Cellular Senescence in Photoaged Skin.” *Scientific Reports* 7 (1): 1–8. https://doi.org/10.1038/s41598-017-15901-9.

Wang, Chunfang, Diana Jurk, Mandy Maddick, Glyn Nelson, Carmen Martin-ruiz, and Thomas Von Zglinicki. 2009. “DNA Damage Response and Cellular Senescence in Tissues of Aging Mice.” *Aging Cell* 8 (3): 311–23. https://doi.org/10.1111/j.1474-9726.2009.00481.x.

Wilhelmus, Micha M.M., Wilbert C. Boelens, Irene Otte-Höller, Bram Kamps, Robert M.W. de Waal, and Marcel M. Verbeek. 2006. “Small Heat Shock Proteins Inhibit Amyloid-β Protein Aggregation and Cerebrovascular Amyloid-β Protein Toxicity.” *Brain Research* 1089 (1): 67–78. https://doi.org/10.1016/j.brainres.2006.03.058.

Zhang, Haoyue, Linlin Sun, Kun Wang, Di Wu, Mason Trappio, Celeste Witting, and Kan Cao. 2016. “Loss of H3K9me3 Correlates with ATM Activation and Histone H2AX Phosphorylation Deficiencies in Hutchinson-Gilford Progeria Syndrome.” *PLoS ONE* 11 (12): 1–25. https://doi.org/10.1371/journal.pone.0167454.

**
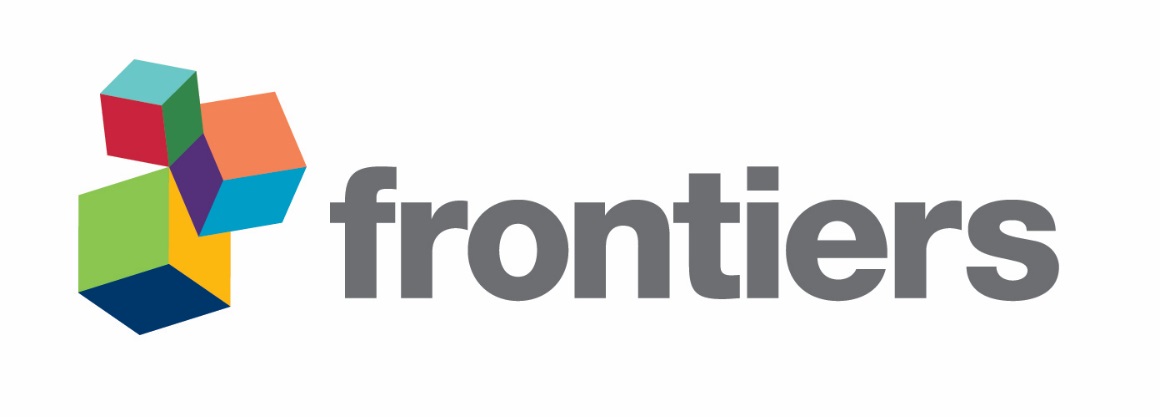
**
